# p62 overexpression induces TDP-43 cytoplasmic mislocalisation, aggregation and cleavage and neuronal death

**DOI:** 10.1038/s41598-021-90822-2

**Published:** 2021-06-01

**Authors:** A. D. Foster, L. L. Flynn, C. Cluning, F. Cheng, J. M. Davidson, A. Lee, N. Polain, R. Mejzini, N. Farrawell, J. J. Yerbury, R. Layfield, P. A. Akkari, S. L. Rea

**Affiliations:** 1 Department of Endocrinology and Diabetes, Sir Charles Gairdner Hospital, Nedlands, WA Australia; 2 Harry Perkins Institute of Medical Research, University of Western Australia, Crawley, WA Australia; 3 Perron Institute for Neurological and Translational Science, Centre for Neuromuscular and Neurological Disorders, The University of Western Australia, Nedlands, WA 6009 Australia; 4 Centre for Molecular Medicine and Innovative Therapeutics, Murdoch University, Health Research Building, Discovery Way, Murdoch, WA 6150 Australia; 5 Department of Biomedical Sciences, Macquarie University, Sydney, Australia; 6 School of Biological Sciences, University of Wollongong, Wollongong, 2522 Australia; 7 School of Life Sciences, University of Nottingham, Nottingham, UK

**Keywords:** Cell biology, Genetics, Molecular biology, Neuroscience

## Abstract

Amyotrophic lateral sclerosis (ALS) and frontotemporal lobar degeneration (FTLD) that exist on a spectrum of neurodegenerative disease. A hallmark of pathology is cytoplasmic TDP-43 aggregates within neurons, observed in 97% of ALS cases and ~ 50% of FTLD cases. This mislocalisation from the nucleus into the cytoplasm and TDP-43 cleavage are associated with pathology, however, the drivers of these changes are unknown. p62 is invariably also present within these aggregates. We show that p62 overexpression causes TDP-43 mislocalisation into cytoplasmic aggregates, and aberrant TDP-43 cleavage that was dependent on both the PB1 and ubiquitin-associated (UBA) domains of p62. We further show that p62 overexpression induces neuron death. We found that stressors (proteasome inhibition and arsenic) increased p62 expression and that this shifted the nuclear:cytoplasmic TDP-43 ratio. Overall, our study suggests that environmental factors that increase p62 may thereby contribute to TDP-43 pathology in ALS and FTLD.

## Introduction

Amyotrophic lateral sclerosis (ALS) and frontotemporal lobar degeneration (FTLD) are genotypically, phenotypically and pathologically related neurodegenerative diseases that are now referred to as part of a disease spectrum^1^. ALS, the most common form of motor neuron disease, is a progressive and ultimately lethal disorder with an average life expectancy of 2–3 years post diagnosis^2^. FTLD is a leading cause of early onset dementia. Although most cases (~ 90%) of both diseases are sporadic (having no known familial involvement), the pathology and phenotypic presentation of sporadic cases are, for the most part, indistinguishable to inherited forms. The exact aetiology of ALS and FTLD remains unclear, however, research over the past few decades indicates that several mechanisms may play a role in pathobiology. These include dysfunctional protein homeostasis through changes to autophagy or lysosomal function or the ubiquitin–proteasome system, endoplasmic reticulum stress, dysfunctional mitochondria and/or energy metabolism, increased oxidative stress, impaired axonal transport and trafficking defects and dysfunctional RNA-binding proteins^2,3^.

A key characteristic of ALS and FTLD is the presence of cytoplasmic aggregates containing TDP-43 in neurons, present in 97% of ALS cases, familial and sporadic, and ~ 50% of FTLD cases, indicating a common final pathway^4^. TDP-43 aggregates are also found in other neurodegenerative and neuromuscular conditions (e.g. Alzheimer’s disease and inclusion body myositis). Mutations in *TARDBP*, coding for TDP-43, are a rare cause of ALS and FTLD^5,6^. In familial and sporadic cases, the TDP-43 observed in affected tissue is found in cytoplasmic aggregates and is aberrantly phosphorylated, ubiquitinated and cleaved^7^. TDP-43 is normally a primarily nuclear protein, with roles in mRNA translation, splicing and cryptic exon retention, nuclear export, and axon outgrowth^4^. The cytoplasmic mislocalisation of TDP-43 is considered an important pathogenic mechanism in ALS and FTLD that is likely to impact diverse biological processes that reduce neuronal viability^4^. TDP-43 pathology may be due to both loss and gain of function^8–10^. Abnormal nuclear depletion of TDP-43 causes a loss of splicing function in neurons leading to widespread transcriptome changes, including altered splicing^11^ and also leads to defects in DNA double-strand repair^12^. TDP-43 cytoplasmic aggregation and cleavage leads to toxic gain of function phenotypes^13^.

TDP-43 positive protein aggregates in ALS and FTLD often also contain p62 protein^14–17^. The consistent presence of p62 in pathological inclusions and the identification of Sequestosome1/p62 mutations as a rare cause of ALS and FTLD suggest a role in pathogenesis^18^. p62 shuttles ubiquitinated proteins to the proteasome or to the autophagy-lysosomal system for degradation and regulation of signalling pathways that are induced by oxidative stress (Nrf2) and nerve growth factor (nuclear factor kappa b, NF-κB). p62 facilitates the degradation of proteins implicated in neurodegenerative diseases, including mutant superoxide dismutase 1 (SOD1) in ALS^19^ and tau in Alzheimer’s, and FTLD^20^. Further to roles in autophagy and the ubiquitin proteasome system the adaptor protein regulates transcription factors. p62 is a scaffold protein in NF-κB signalling pathways important for neuronal differentiation and survival^21^. Oxidative damage to the p62 promoter is associated with neurodegenerative diseases, p62 knockout mice develop dementia-like symptoms, and loss of p62 caused a syndrome that includes childhood neurodegeneration^22,23^. Together, these studies suggest that increasing p62 levels may be of therapeutic benefit. However, previous studies highlight that p62 knockout can enhance neuron survival or that p62 expression can be detrimental^24,25^. It remains unclear whether p62 expression is harmful or helpful in the context of ALS and FTLD. Here, we describe that overexpression of p62 leads to TDP-43 cellular mislocalisation, cleavage and aggregation and leads to neuronal death.

## Results

### Over-expression of p62 causes mislocalisation and nuclear depletion of wild type TDP-43

**Figure 1:**
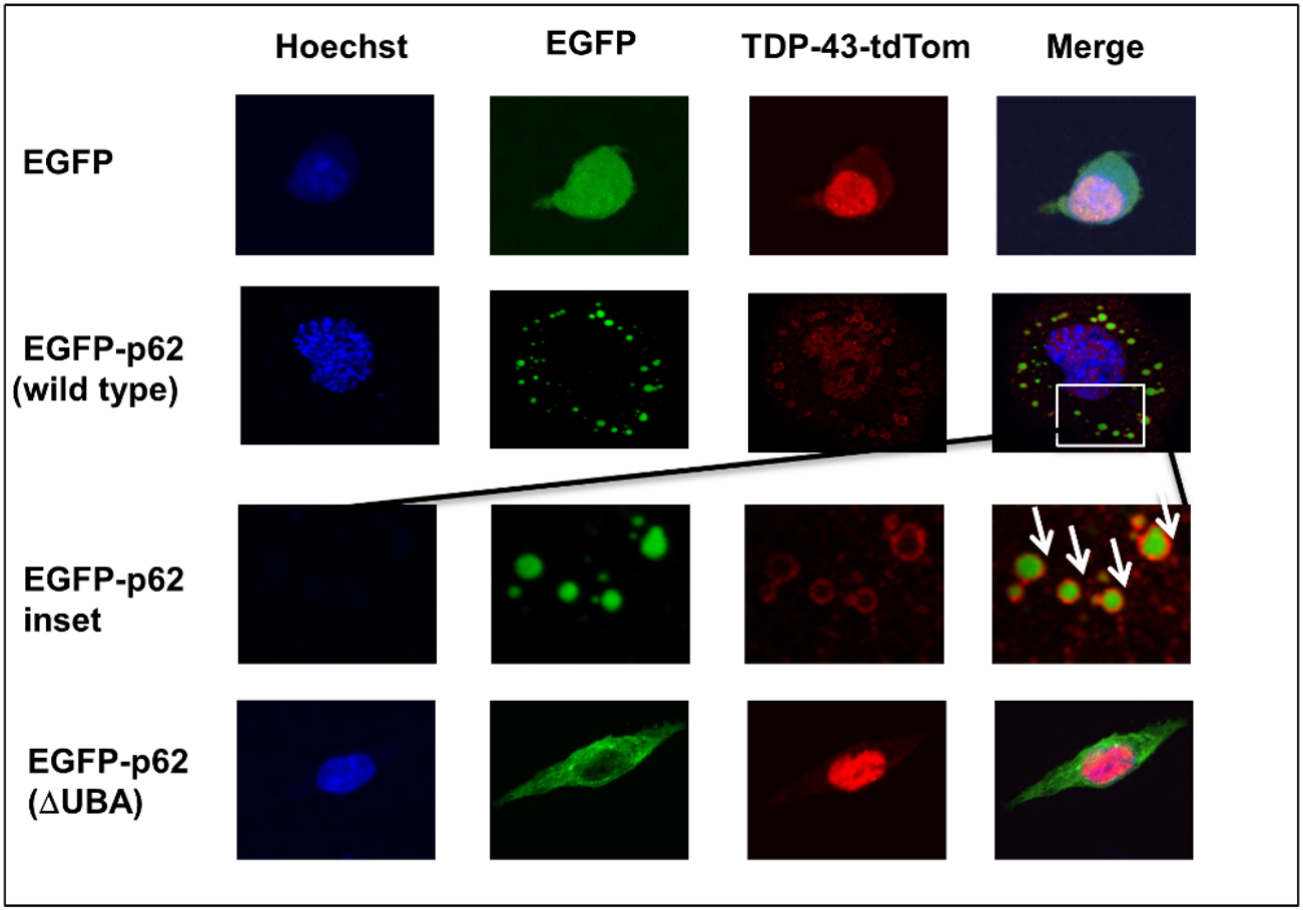
Expression of EGFP-p62 causes TDP-43^WT^ cytoplasmic mislocalisation in a UBA domain dependent manner. Cells co-expressing TDP-43^WT^ and either EGFP or EGFP-p62 (WT or ΔUBA) were fixed and stained with Hoechst then visualized by confocal microscopy. Arrows show TDP-43 forming a shell around wild type p62 (inset). Three independent experiments were performed. Cells expressing both TDP-43-tdTomato and EGFP construct were imaged from random fields of view with a minimum of 10 cells imaged per experiment. TDP-43-tdTomato co-localisation with the nucleus was determined by calculating the Pearson’s co-efficient with ImageJ. Post-hoc ANOVA Bonferroni test was performed in SPSS.

TDP-43 is the main component of protein aggregates in ALS and non-tau related FTLD. p62 is invariably also observed in cytoplasmic protein aggregates within surviving neurons in neurodegenerative diseases including ALS and FTLD, and p62 and TDP-43 were co-immunoprecipitated from FTLD-affected tissue^17^. Thus, we wondered if p62 expression or its aggregation within the cytoplasm might affect TDP-43 localisation. We observed that overexpression of EGFP-p62^WT^ with TDP-43-tdTomato^WT^ in motor neuron-like NSC-34 cells causes TDP-43 nuclear depletion with localisation shifting into cytoplasmic aggregates. Co-localisation of TDP-43 with the nuclear stain was significantly reduced in cells expressing EGFP-p62^WT^ compared with those expressing EGFP alone (*p* =  < 0.001) with Pearson’s co-efficient of 0.508 ± 0.047 versus 0.786 ± 0.021. (Fig. 1). A Pearson’s co-efficient of > 0.6 indicates co-localisation, thus p62 overexpression significantly reduced TDP-43 co-localisation with the nucleus. We investigated the effects of increasing concentrations of EGFP-p62^WT^ by transfecting cells with 0.5, 1 or 2 reflect µg of expression construct and observed that all concentrations led to TDP-43-tdTomato^WT^ mislocalisation and cytoplasmic aggregation (Supplementary Fig. 1).

As p62 and TDP-43 often co-localise with ubiquitin in ALS and FTLD tissue, and p62 recognises ubiquitinated proteins via its ubiquitin-associated (UBA) domain, we investigated whether nuclear mislocalisation of TDP-43 was dependent on the UBA domain. Of note, several *SQSTM1*/p62 mutations reported in ALS and FTLD patients manifest within the UBA domain (Reviewed in^18^) and have previously been shown to reduce ubiquitin-binding capacity^26^. We found that co-expression of EGFP-p62^ΔUBA^ with TDP-43-tdTomato did not lead to mislocalisation and nuclear depletion of TDP-43, as co-localisation with the nucleus was unaffected by EGFP-p62^ΔUBA^ over-expression (Pearson’s co-efficient of 0.651 ± 0.036). Of note, we observed that TDP-43 in the cytoplasm formed a distinct shell surrounding EGFP-p62^WT^ (Fig. 1, inset, arrows), this phenomenon was not observed with co-expression of EGFP-p62^ΔUBA^.

### p62 shifts TDP-43 solubility in an UBA-dependent manner

**Figure 2:**
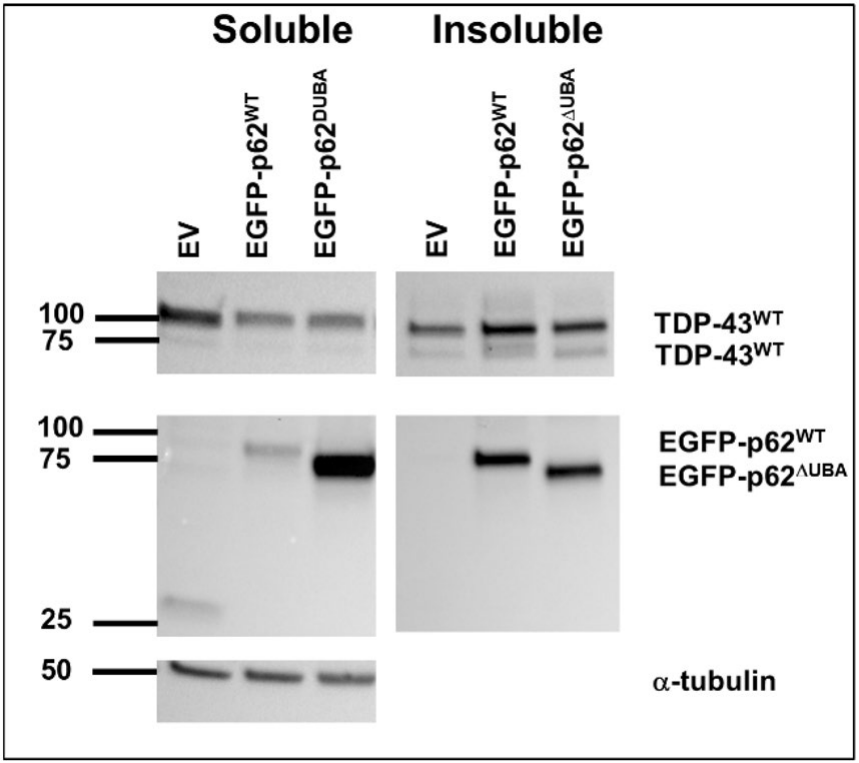
p62 expression alters TDP-43 solubility. Cells were transfected with TDP-43^WT^ and either EGFP, EGFP-p62^WT^ or EGFP-p62^ΔUBA^ (as indicated), and 48 h post-transfection cells were lysed in Triton-X buffer (soluble). Triton-X insoluble pellets were solubilized with SDS (insoluble). Western blot analyses with GFP, RFP and α-tubulin antibodies were performed. Image is representative of 3 independent experiments.

We observed that TDP-43 mislocalisation from the nucleus seemed to be associated with p62 foci, as TDP-43 visually formed a shell around p62 puncta. Thus, we performed fractionation of cellular proteins into a soluble and an insoluble (containing protein aggregates) fraction to determine the effects of p62 on TDP-43 aggregation (Fig. 2).

Consistent with the visual assessment of p62 puncta that we observed in our confocal experiments, EGFP-p62^WT^ was predominantly present in the insoluble fraction, whereas EGFP-p62^ΔUBA^ was predominantly present in the soluble fraction. This fits with our observations that the EGFP-p62^ΔUBA^ mutant was principally expressed in a diffuse pattern within the cytoplasm. We found that co-expression with EGFP-p62^WT^, but not EGFP-p62^ΔUBA^, decreased the soluble:insoluble ratio of wild type TDP-43 (*p* = 0.01); which appears to be due to the decreased soluble TDP-43 in cells, EGFP-p62^WT^ *p* = 0.080 (Fig. 2). The total TDP-43 was not significantly different in EGFP-p62 (WT or ∆UBA) expressing cells compared to those transfected with EGFP control cells, suggesting that p62 expression did not affect turnover of TDP-43^WT^ (Table 1).

**Table 1:** Expression of EGFP-p62 reduces TDP-43 solubility in a UBA-dependent manner.

	Soluble	Insoluble	Sol:Insol	Total
EGFP-p62^WT^	↓ *p* = 0.080	n.s	↓ *p* = 0.01	n.s
EGFP-p62^∆UBA^	n.s	n.s	n.s	n.s

### Nuclear TDP-43 is significantly decreased in p62^+/+^ compared with p62^−/−^ cells

**Figure 3:**
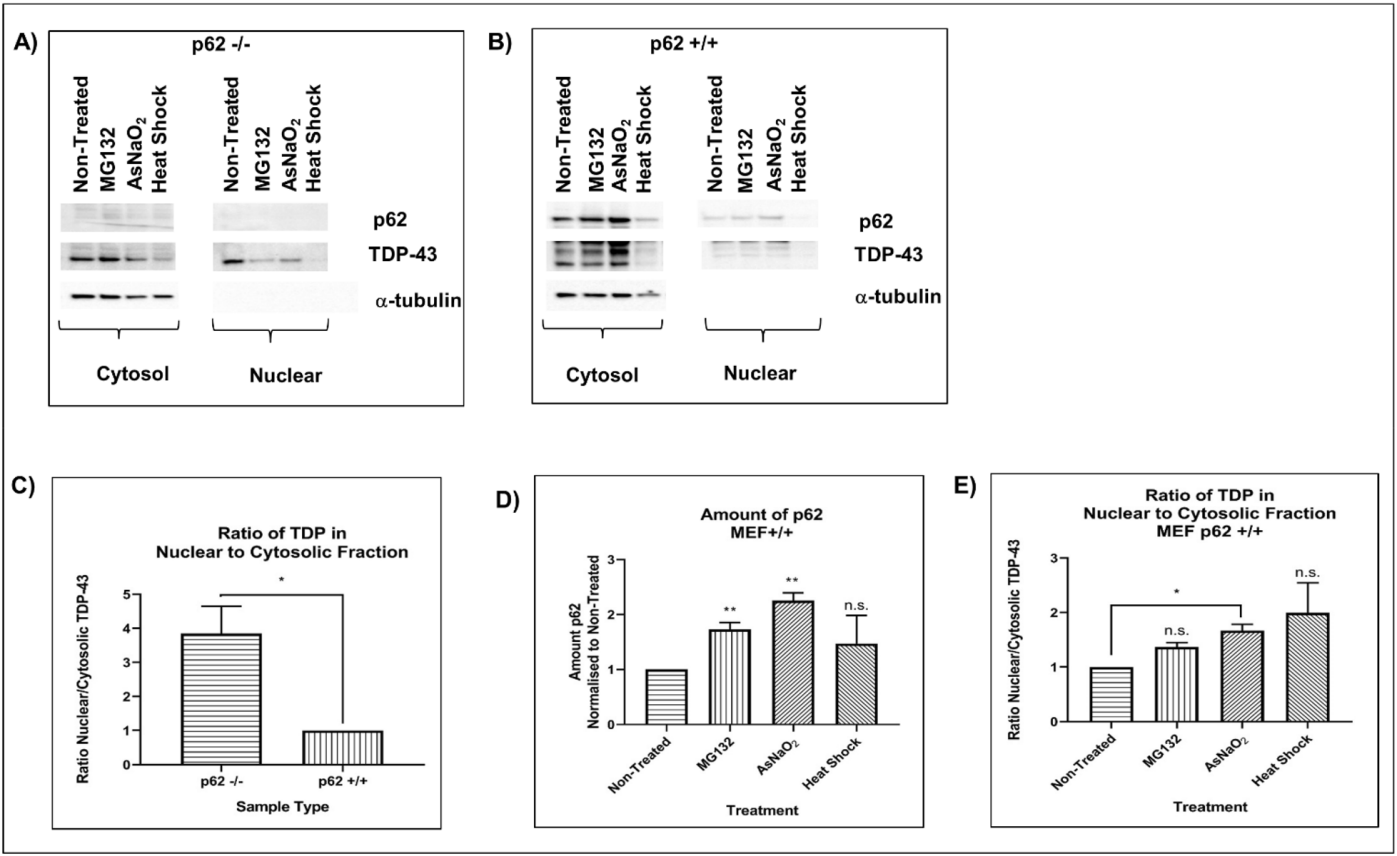
p62 alters the nuclear:cytoplasmic TDP-43 ratio. Mouse embryonic fibroblasts (**A**) with p62 knockdown (p62^−/−^) and (**B**) without (p62^+/+^) were treated with proteasome inhibitor (MG132), sodium arsenate or heat shock followed by recovery. Cells were fractionated into nuclear and cytosol fractions and proteins separated by SDS polyacrylamide gel electrophoresis. Western blots for endogenous p62, TDP-43 and α-tubulin were performed. (**C**) The ratio of nuclear: cytoplasmic TDP-43 in non-treated p62^−/−^ cells presented as a fold-change normalised to p62^+/+^ non-treated cells (set to 1.0) and (**D**) the amount of p62 in p62^+/+^ with indicated treatments. (**E**) The ratio of nuclear:cytoplasmic TDP-43 in treated p62^+/+^ cells presented as a fold-change normalised to p62^+/+^ non-treated cells (set to 1.0). Images are representative of 4 independent experiments. Data presented in (**C**), (**D**) and (**E**) are the mean +/− SEM of 4 independent experiments.

We confirmed our finding that p62 expression is an important factor that regulates TDP-43 localisation using mouse embryonic fibroblasts (MEFs) with (p62^+/+^) and without p62 (p62^−/−^). The amount of nuclear TDP-43 was increased in p62^−/−^ cells compared with p62^+/+^, thus increasing the nuclear:cytoplasmic ratio (Fig. 3A–C). Because p62 aggregates are observed in ALS, FTLD and other neurodegenerative disorders, we hypothesised that environmental risk factors for these diseases may increase p62 expression and this could lead to TDP-43 cytoplasmic mislocalisation. In support of this hypothesis, previous literature shows that head trauma, which may be a risk factor for ALS and FTLD^27–29^, increases p62 expression leading to p62/TDP-43 aggregation in neurons^30,31^. To confirm the role of p62 in TDP-43 localisation we used p62^+/+^ and p62^−/−^ MEFs. We treated cells with a proteasome inhibitor (MG132), sodium arsenate (NaAsO2) or heat shock and observed that MG132 and arsenic both increased p62 expression in p62^+/+^ cells (Fig. 3D). This was associated with an increase in the amount of cytoplasmic TDP-43 observed (Fig. 3E). While MG132 increased the level of cytoplasmic TDP-43 in p62^−/−^ MEFs, NaAsO_2_ did not, indicating that p62 is required for arsenate but not MG132-induced TDP-43 mislocalisation.

### p62 overexpression induces TDP-43 cleavage to create a disease-associated 35-kDa fragment

**Figure 4:**
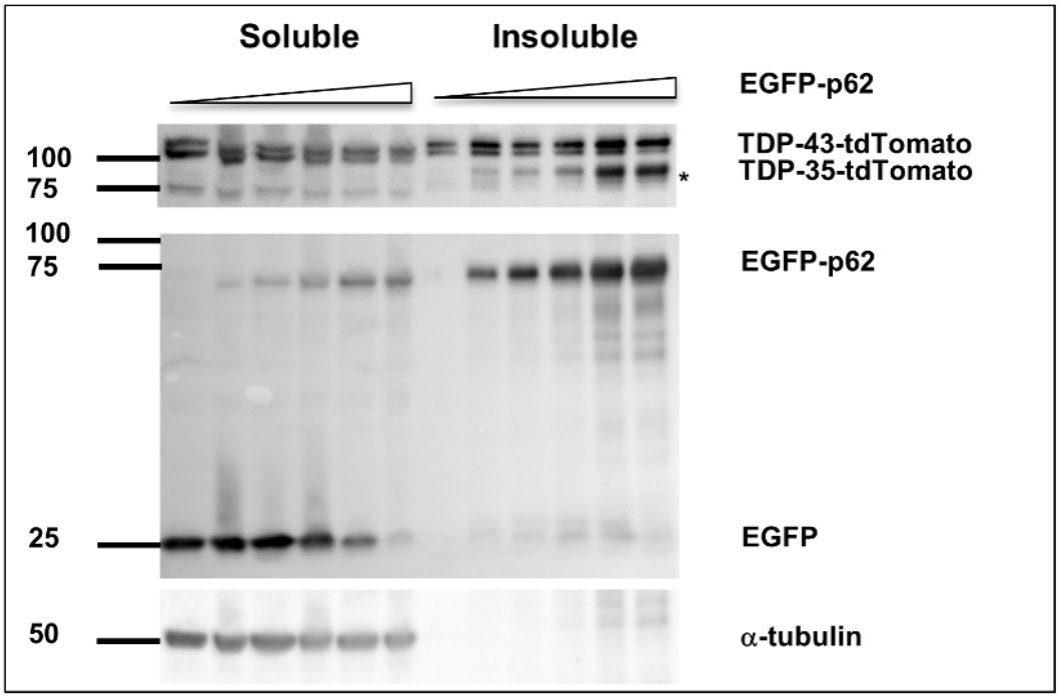
p62 expression induces TDP-43 cleavage to a 35-kDa fragment. Cells were transfected with constant TDP-43-tdTomato with increasing amounts of EGFP-p62 (0, 0.25, 0.5, 1, 1.5 or 2 μg) and decreasing amounts of EGFP (2, 1.75, 1.5, 1, 0.5 or 0 μg) to maintain consistent DNA amounts across transfection. Cells were then lysed in Triton-X buffer (soluble) and Triton-X insoluble pellets were solubilized with SDS (insoluble). Western blots for GFP, RFP and α-tubulin were performed as indicated. Image is representative of 3 independent experiments.

Cleaved TDP-43 products of 35- and 25-kDa have been identified in ALS and FTLD^28^. Aggregation of the 35-kDa fragment, but not the 25-kDa fragment, induced cytoplasmic inclusions that recruited full length TDP-43 and inhibited RNA processing^32^. We investigated the effect of increasing p62 expression on TDP-43 aggregation and cleavage. We observe that as p62 overexpression is increased, more TDP-43 becomes insoluble. Further, we observed the production of a cleaved 90-kDa TDP-43-tdTomato fragment (Fig. 4). As tdTomato is ~ 55 kDa and is a C-terminal tag in this construct, this fragment presumably corresponds to a cleaved C-terminal 35-kDa product of TDP-43 (TDP-35).

### Proteasome inhibition and p62 overexpression induce cleavage of TDP-43

**Figure 5:**
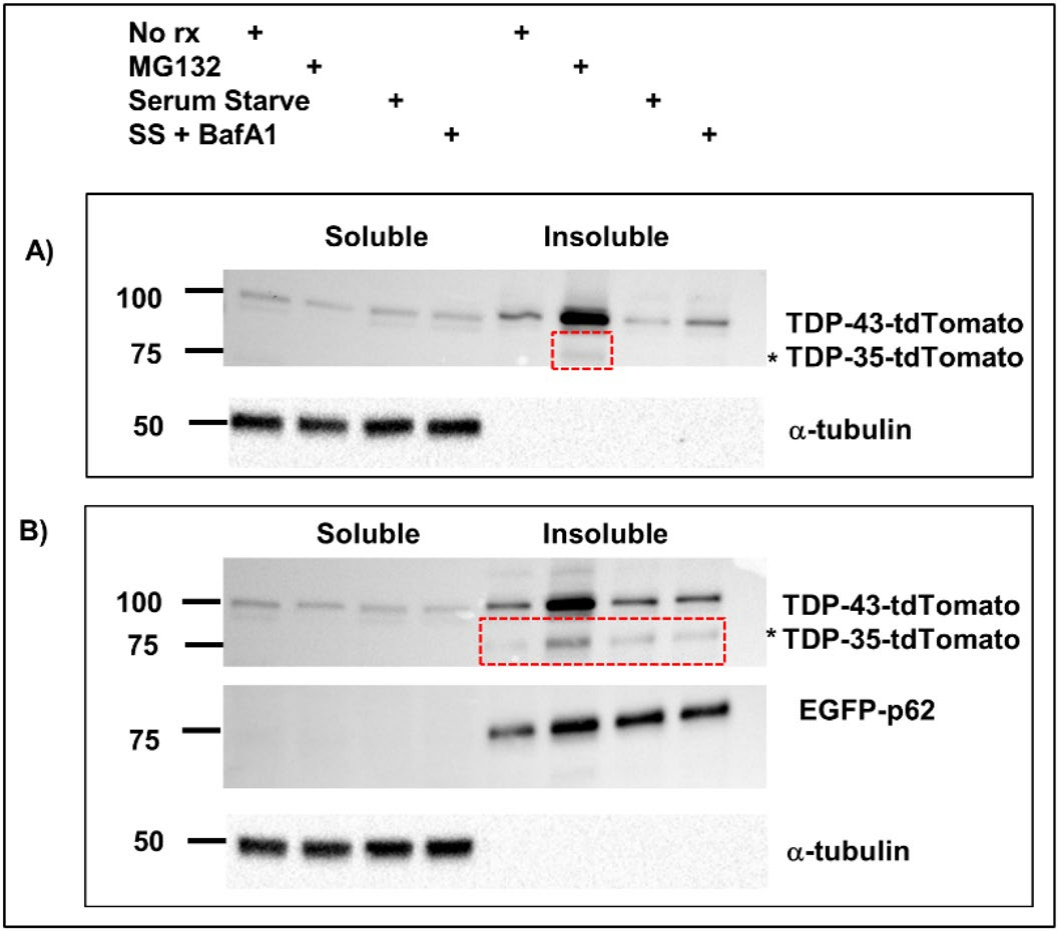
p62 creates a cleaved TDP-43 species that is also induced by proteasomal inhibition. Cells were transfected with TDP-43-tdTomato and either pcDNA3.1 (**A**) or EGFP-p62 (**B**). Cells were treated with proteasome inhibitor MG132, serum starved, serum starved with Bafilomycin A1 or left untreated. Cells were lysed and soluble and insoluble fractions obtained. Western blots were performed as indicated. Red dashed boxes indicate the presence of a *TDP-43-tdTomato cleavage product (TDP-35-tdTomato). The TDP-35 band from pcDNA3.1 transfected MG132-treated cells and EGFP-p62 non-treated cells was excised, trypsin-digested and analysed by mass spectrometry (Fig. 6). However, we did observe that the presumed TDP-35 fragment induced by EGFP-p62^WT^ overexpression was also present in the lysates from EGFP expressing cells that were treated with the MG132. These protein bands were excised for LC–MS/MS analysis and we identified four tryptic peptides to confirm that the band matching 90-kDa TDP-tdTomato was indeed a cleavage product of TDP-43 (Fig. 6).

Cleavage of TDP-43 can be mediated by caspase-3 or calpains and may be a precursor to its degradation. TDP-43 can be degraded by either the proteasome or the autophagy-lysosomal pathway with p62 reportedly targeting TDP-43 to both^33^. As this study suggested that p62 mediates TDP-43 degradation, we further investigated whether p62-induced TDP-43 cleavage was associated with TDP-43 degradation. We co-expressed TDP-43^WT^ with EGFP-p62^WT^ and 24 h post-transfection treated cells with MG132, serum starved cells to induce autophagy or serum starved and Bafilomycin A1 treated cells to inhibit lysosomal degradation. We did not see any significance differences in the amount of total TDP-43, a combination of that present in the soluble and insoluble fractions (full-length and cleaved), in cells overexpressing EGFP-p62^WT^ compared with cells overexpressing EGFP. Thus, in the time frame of our experiment 48 h post-transfection EGFP-p62^WT^ overexpression, and the subsequent cleavage of TDP-43 to TDP-35, did not enhance TDP-43 degradation (Fig. 5).

**Figure 6:**
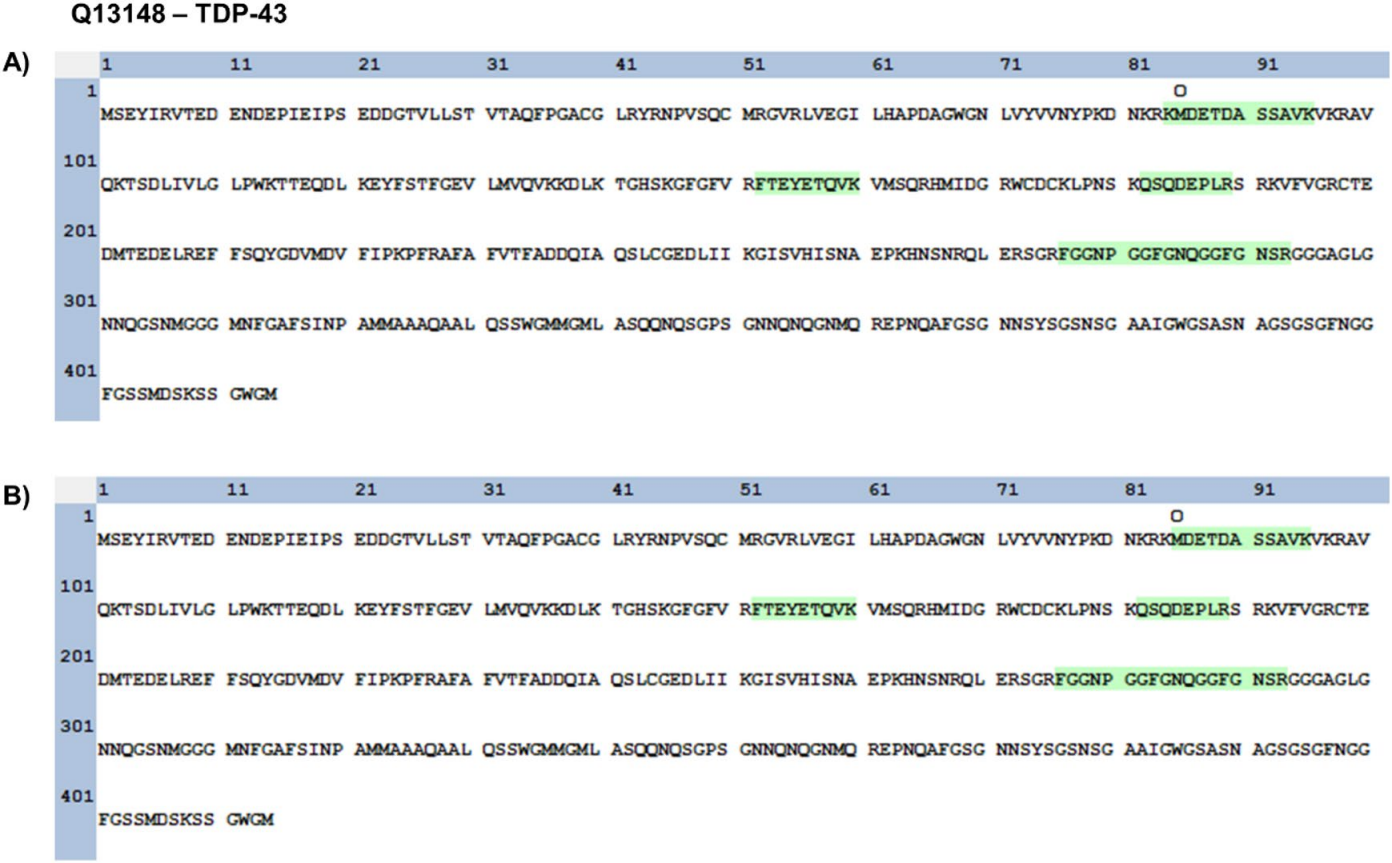
Identified tryptic peptides from TDP-43. The 90 kDa-tdTomato-TDP43 bands from cells transfected with the empty vector treated with MG132 (**A**) and overexpressed with p62 (**B**) were excised and underwent tryptic digestion. Peptide analysis indicated that the fragments consist of the C-terminal aa85-414 of TDP-43.

These four tryptic peptides of TDP-43 mapped to regions corresponding to amino acids 85–414 (C-terminal region) of TDP-43, indicating that the N-terminal region of TDP-43 was presumably cleaved (Supplementary Figs. 5–8).

**Figure 7:**
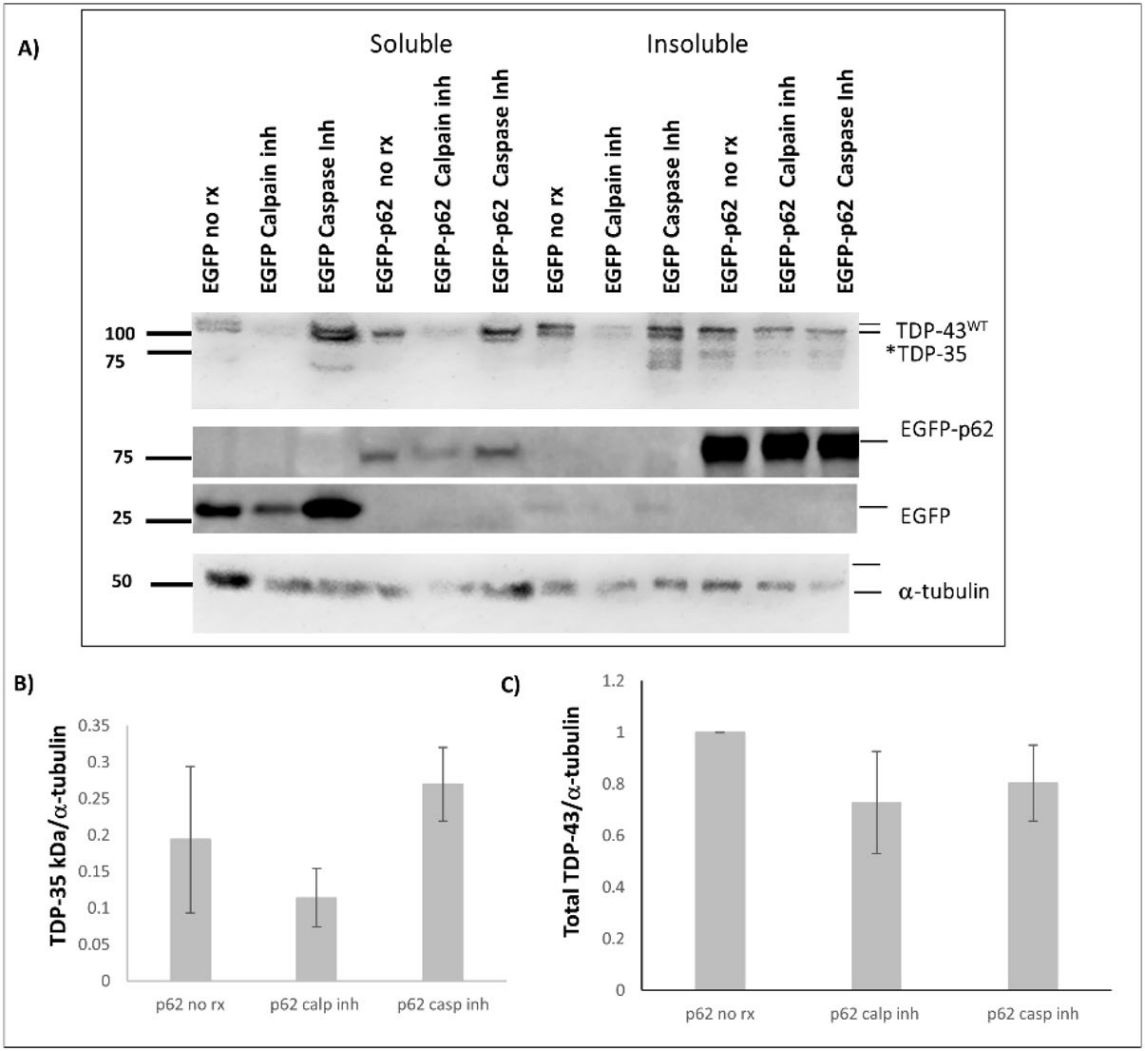
p62-mediated cleavage of TDP-43 is independent of caspases. NSC-34 cells were pre-treated with 20 μM caspase inhibitor, 5 μM calpain inhibitor or were left untreated for 1 h and then transfected with TDP-43-tdTomato and either EGFP or EGFP-p62. Cells were maintained in media containing the inhibitor for 48 h and then harvested for soluble and insoluble fractions. Western blot analyses for TDP-43 (anti-RFP), EGFP and α-tubulin were performed. TDP-43 bands were quantified using ImageJ and the amount of TDP-35-tdTomato fragment observed in the insoluble fraction of EGFP versus EGFP-p62 expressing cells was compared with SPSS.

As a control, we also excised a region on the protein gel corresponding to where endogenous TDP-43 migrates from HEK293 lysate and identified predominately tryptic peptides that mapped to the N-terminal region of TDP-43 (Supplementary Fig. 9). This suggests that the band matching 90-kDa TDP-43-tdTomato (TDP-35-tdTomato) was cleaved at the N-terminus, which possibly could be attributed to a Caspase-3 dependent “DETD” recognition site at amino acids 85–89^30^. TDP-43 is also cleaved by calpains, therefore we next investigated whether the cleavage was in fact dependent on caspases or calpains using inhibitors. Cells were pre-treated with caspase inhibitor or a calpain inhibitor prior to transfection with expression constructs for TDP-43-tdTomato and either EGFP or EGFP-p62. Cells were maintained with the inhibitors for 48 h and then subjected to fractionation. The amount of TDP-35-tdTomato observed in the insoluble fraction of EGFP-p62 expressing cells treated with caspase inhibitor was not significantly different to that in the lysates obtained from non-treated cells (Fig. 7). The amount of TDP-35-tdTomato in calpain inhibitor treated EGFP-p62 expressing cells was lower than was present in non-treated cells, however this is likely a reflection of reduced total TDP-43.

### p62 overexpression induces neuronal cell death

**Figure 8:**
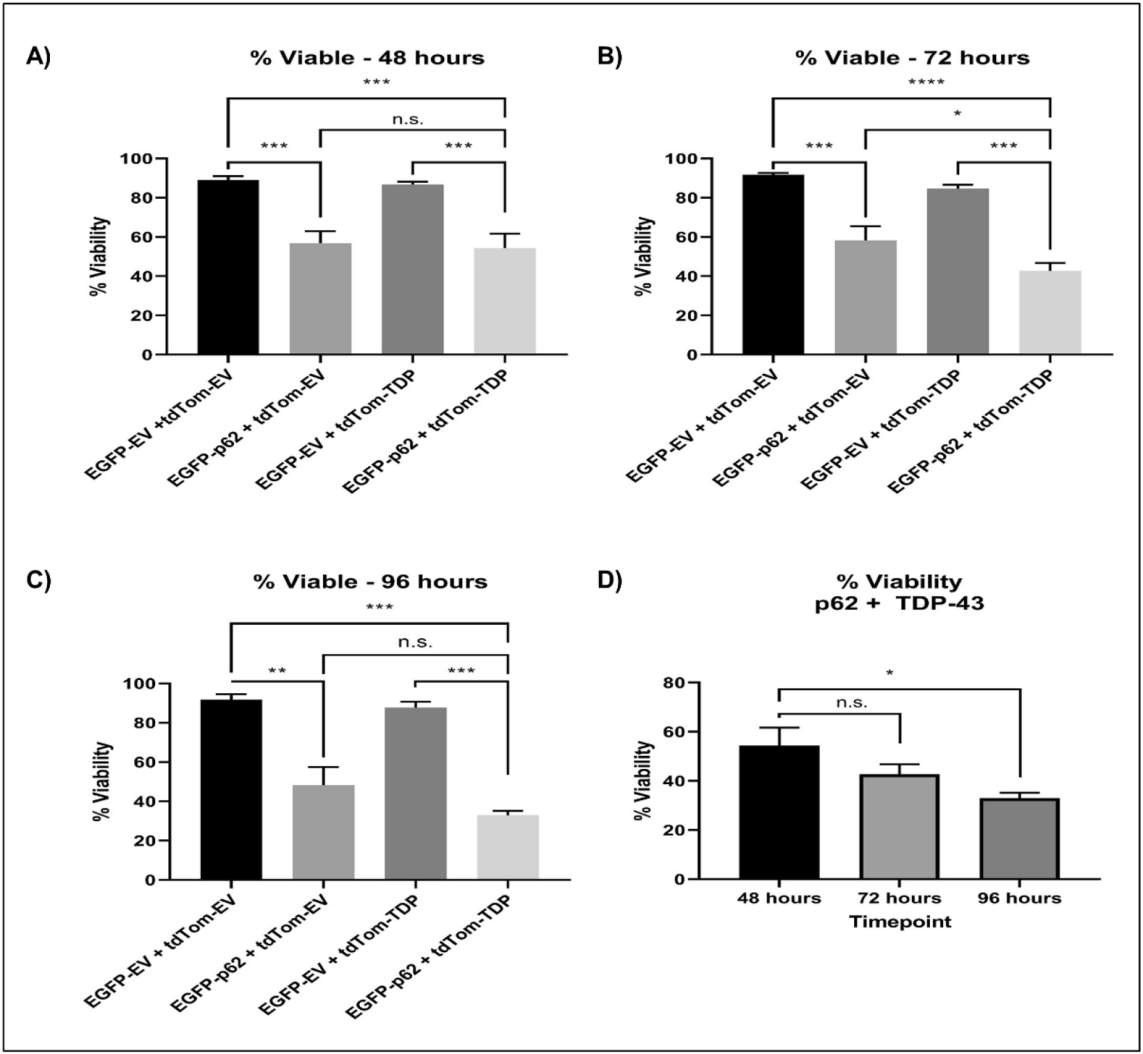
Expression of p62 causes neuronal death. Cells were transfected with EGFP empty vector (EGFP-EV) and RFP empty vector (RFP-EV), EGFP-p62 with or without TDP-43-tdTomato (RFP-TDP) or TDP-43-tdTomato with EGFP-EV. At (**A**) 48, (**B**) 72 or (**C**) 96 h post-transfection cells were trypsinised and incubated with Sytox-Red and assessed for cell viability by flow cytometry, (**D**) percentage viability of cells expressing EGFP-p62 + RFP-EV compared to EGFP-EV + RFP-EV expressing cells at each time point. Data is the mean of 3 independent experiments +/− SEM.

While previous studies have shown that p62 knockdown causes neurodegeneration^22,23^, overexpression of p62 in an ALS SOD1 mouse exacerbated disease^24^. Because we have observed that p62 overexpression leads to TDP-43 mislocalisation, aggregation and cleavage, known signs of pathology in ALS and FTLD, we hypothesised that p62 overexpression may be detrimental to neurons. Therefore, we performed cell viability assays. NSC-34 cells were transfected with tagged-empty vector controls or EGPP-p62^WT^ with TDP-43^WT^ or with tdTomato empty vector control. We assessed cell viability at 48, 72 and 96 h post transfection and observed significant cell death at each time point (Fig. 8). The percentage of viable cells decreased from ~ 90% in cells expressing controls to 56.83% (*p* = 0.0009), 58.27% (*p* = 0.0004) and 48.3% (*p* = 0.0018) with EGFP-p62^WT^ expression alone at 48, 72 and 96 h post transfection, respectively. In cells expressing both TDP-43^WT^ and EGFP-p62^WT^ this was further reduced to 42.73% (*p* < 0.0001) and 32.97% (*p* = 0.0004) at 72 and 96 h post transfection, respectively.

**Figure 9:**
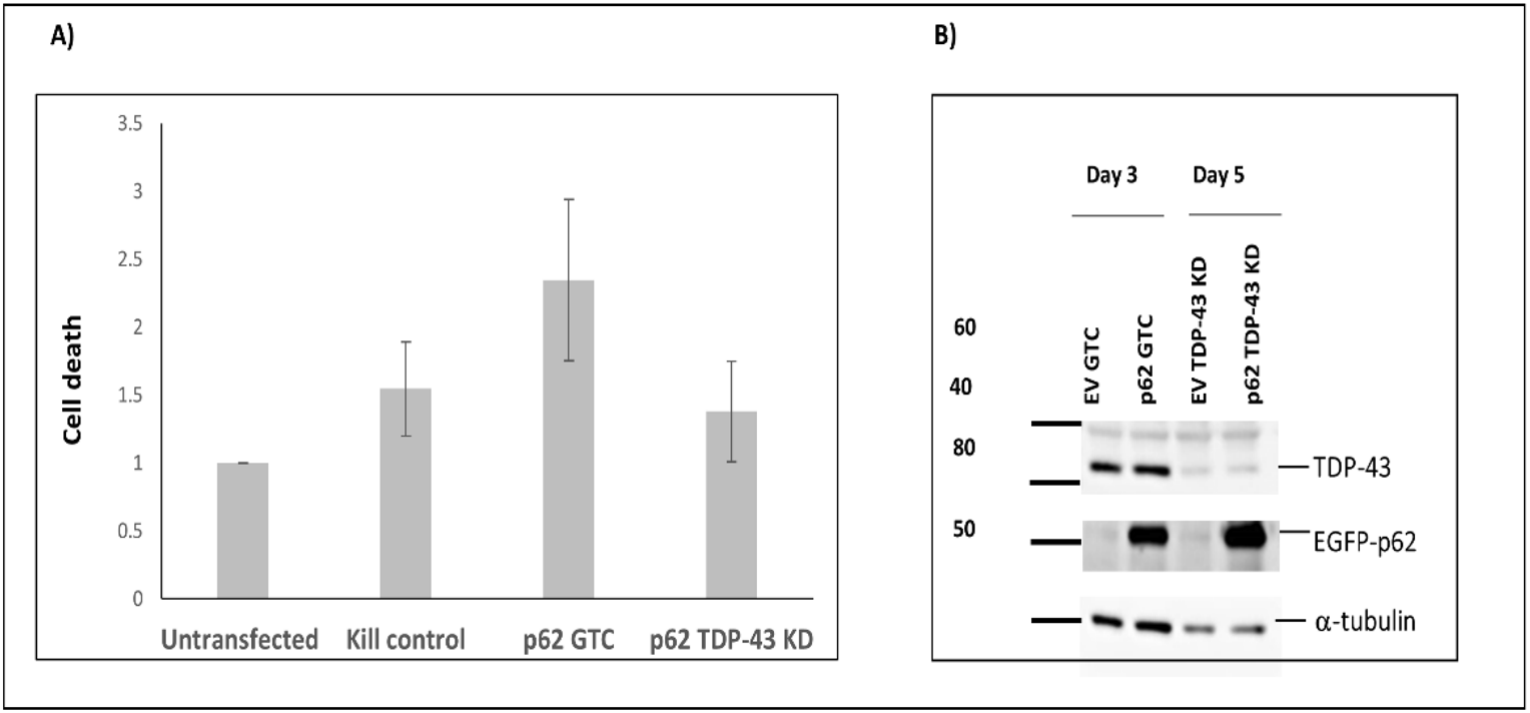
p62-mediated neuronal death is independent of TDP-43 expression. (A) Cells were transfected with a PMO to knock-down TDP-43 (TDP-43 KD) or a standard control (GTC). The following day cells were transfected with EGFP-p62 and 48 h later trypsinised, incubated with Sytox-Red and then assessed for cell viability by flow cytometry. (B) Cells were harvested at 3 and 5 days post transfection with PMOs and western blots performed as indicated.

Next we investigated whether the effects of p62 overexpression on cell viability were dependent on TDP-43. To this end, we transfected cells with phosphorodiamidate morpholino oligomers (PMOs) used routinely in our laboratory to knock-down expression of TDP-43, or a control PMO (Fig. 9). We found that the toxic effects of overexpressing p62 was not significantly different between cells transfected with TDP-43 knock-down PMOs compared with control PMO transfected cells.

### p62-mediated depletion of nuclear TDP-43 alters RNA splicing and regulation.

**Figure 10:**
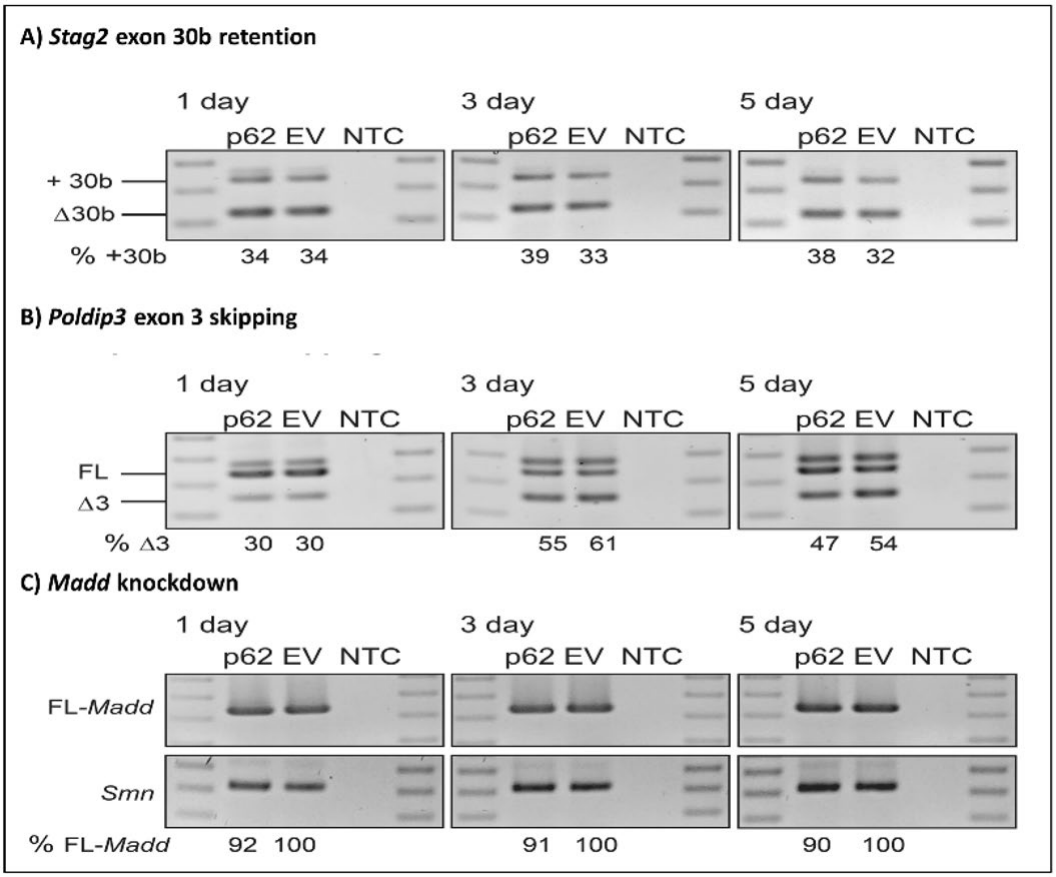
p62 overexpression leads to loss of TDP-43-mediated RNA regulation. Effects of p62 overexpression on alternative splicing of selected genes, showing RT-PCRs across *Stag2* (**A**), *Poldip3* (**B**) and *Madd* (**C**) transcripts following transfection with p62 overexpression vector (p62) or the empty vector control (EV) and incubated for 1, 3 and 5 days. An RT-PCR across *Smn* transcripts were used as a loading control. A 100 bp ladder was loaded on either side of the gel and a no template control (NTC) was included in the final lane. Full-length (FL) and exon skipped (Δ) transcripts are annotated and the percentage of specific transcripts are detailed below each lane.

We wanted to know the effects of p62 overexpression on TDP-43 mediated RNA regulation. To determine whether p62-mediated TDP-43 cleavage and cytoplasmic aggregation leads to a loss of functional nuclear TDP-43, we evaluated the pre-mRNA splicing and regulation of known TDP-43 RNA targets. RT-PCR across the *Stag2*, *Poldip3*, and *Madd* transcripts was carried out on RNA from NSC-34 cells transfected with the p62 overexpression plasmid or an empty vector control, following a 1, 3 or 5-day incubation (Fig. 10). There was a 6% increase in *Stag2* transcripts containing exon 30b (+ 30b) in the p62 transfected cells after 3 and 5 days compared with empty vector transfected cells, with 39% of + 30b transcripts compared to 33% within the empty vector transfected cells (Fig. 10A). We observed a decrease in the number of transcripts skipping exon 3 (Δ3) in *Poldip3* following p62 overexpression, with 47% exon 3 skipping compared to 54% in the empty vector control after 5 days (Fig. 10B). An amplicon above the full length *Poldip3* (FL) transcript was observed and deemed to be a PCR artefact. Following p62 overexpression, we observed up to a 10% decrease in the full-length *Madd* transcript after 5 days, when normalised to *Smn* loading (Fig. 10C).

### The PB1 and UBA domains and the nuclear export sequence of p62 are required for TDP-43 cleavage

**Figure 11:**
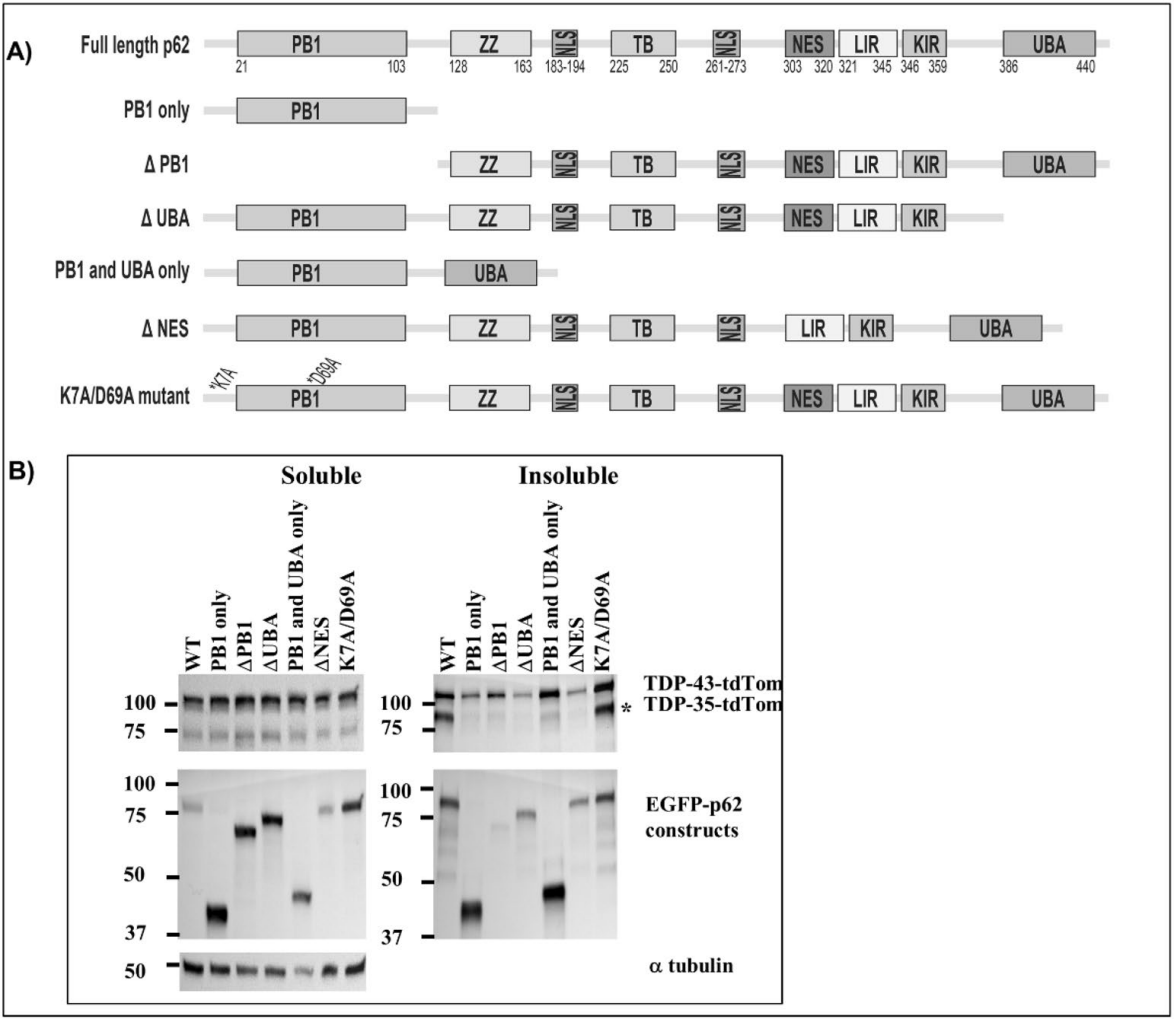
TDP-43 aggregation and cleavage require the PB1 and UBA domains, and the nuclear export signal of p62. NSC-34 cells were transfected with TDP-43-tdTomato and EGFP-p62 wild type [WT] or mutants as indicated. (**A**) Schematic diagram of p62 domain structure and deletion mutants: 1–122 (PB1 domain only), 124–440 (lacks the PB1 domain), 1–385 (lacks the UBA domain), Δ122–386 (PB1 and UBA domains only), Δ303–320 (lacks the nuclear export sequence) and K7A/D69A (self-dimerization mutant). (**B**) Soluble and insoluble fractions were prepared and separated by SDS-PAGE and transferred to nitrocellulose membranes. Western blot analyses were performed as indicated. Image is representative of 3 independent experiments.

In order to determine which regions of p62 are required for TDP-43 pathology, we co-expressed TDP-43-tdTomato with EGFP-p62 deletion constructs and fractionated cells into soluble and insoluble fractions. We found that overexpression of an EGFP-p62 protein consisting of the PB1 and the UBA domains only (Δ122–386) or expression of a mutant protein incapable of self-dimerisation (K7A/D69A) induced TDP-43 cleavage to a similar extent as expression of EGFP-p62^WT^ (Fig. 11). However, in contrast to expression of EGFP-p62^WT^, expression of EGFP-p62^∆PB1^ (lacking the PB1 domain (124–440), the UBA domain (1–385) or the nuclear export sequence (Δ303–320) did not induce TDP-43 cleavage to a TDP-35 C-terminal fragment (Fig. 8). While expression of the PB1 domain alone (1–122) was not sufficient to induce significant cleavage of TDP-43, expression of a PB1 and UBA domain only construct (∆122–386) did induce some cleavage. Thus, the PB1 domain, the nuclear export sequence and the UBA domain are required for TDP-43 cleavage.

### The PB1 and UBA domains, but not the nuclear export sequence, of p62 are required for TDP-43 cytoplasmic mislocalisation

**Figure 12:**
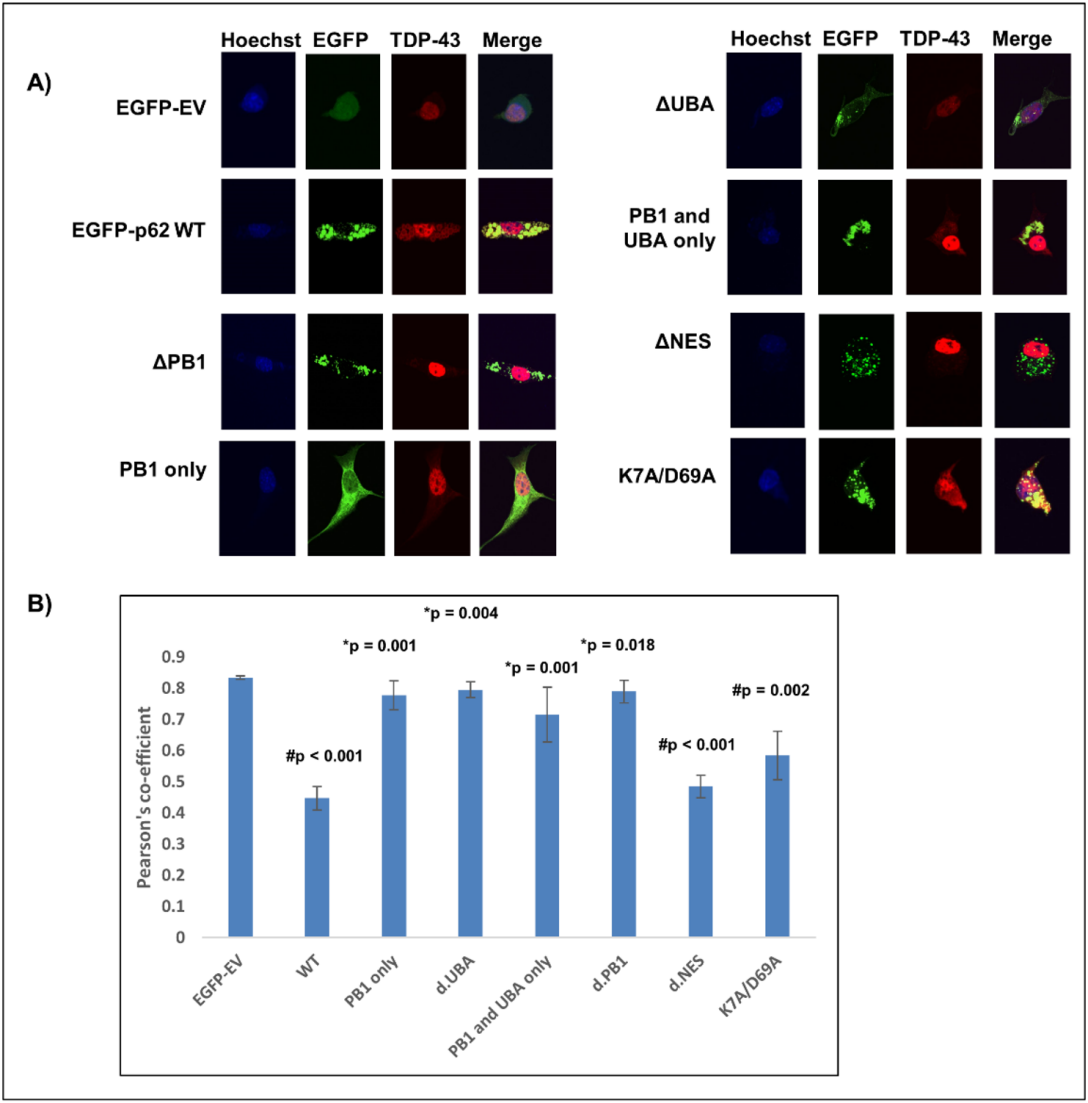
TDP-43 nuclear depletion requires the PB1 and UBA domains of p62, but not the nuclear export signal or self-dimerisation via the PB1 domain. (**A**) NSC-34 cells were transfected with TDP-43-tdTomato and EGFP empty vector (EGFP-EV) or EGFP-p62 (WT or deletion constructions as indicated). Cells were fixed and stained with the nuclear stain Hoechst. Images are representative of 3 independent experiments. Minimum number of cells analysed per construct = 32. (**B**) TDP-43-tdTomato co-localisation with the nucleus was determined by calculating the Pearson’s co-efficient with ImageJ. Statistical analyses were performed using SPSS, with Bonferroni and Dunnet T3, *p* = 0.05. #compared with EGFP-EV, *compared with EGFP-p62 WT.

Overexpression of EGFP-p62^∆PB1^ or EGFP-p62^∆UBA^ failed to mislocalise TDP-43 or cause nuclear depletion, however overexpression of the PB1 and UBA domains alone (EGFP-p62^Δ123–385^) was not sufficient to cause significant TDP-43 nuclear depletion (Fig. 9). We observed that the nuclear export sequence and the self-oligomerisation function of the PB1 domain are not required for TDP-43 mislocalisation to the cytoplasm (Fig. 12). Thus, while the PB1 and UBA domains and the nuclear export sequence were required for TDP-43 cleavage, only the PB1 and UBA domains are required for mislocalisation and p62-induced TDP-43 pathology is not dependent on p62 self-dimerisation.

## Discussion

The mislocalisation of TDP-43 away from the nucleus and into cytoplasmic aggregates in neuronal cells is a hallmark of ALS and non-tau related FTLD. These protein aggregates invariably also contain p62, which is thought to be a marker of dysregulated proteostasis. The cause of protein aggregation and its potential role as either a cytoprotective or cytotoxic process has long been debated and is still unclear^34^. As p62 and TDP-43 are both constituents of these pathogenic cytoplasmic protein aggregates, and co-precipitate from diseased tissue^17^, we wondered if altered p62 expression may affect TDP-43 localisation or aggregation. We show that p62 overexpression causes a shift of TDP-43 out of the nucleus and into the cytoplasm, where it forms a shell-like structure surrounding aggregates of p62. However, overexpression of p62 lacking either the PB1 or the UBA domains failed to induce TDP-43 cytoplasmic mislocalisation or aggregation, suggesting that they are required domains for the induction of TDP-43 pathology. Of note, we show that the self-oligomerisation function of the PB1 domain is not required for mislocalisation to occur. As this function is required for the role of p62 in autophagy, this suggests that the aggregates formed are not destined for protein degradation via this pathway. We confirmed the importance of p62 as a regulator of TDP-43 localisation using p62^−/−^ MEFs. The amount of nuclear TDP-43 and the nuclear:cytoplasmic ratio was increased in these cells compared to wild type counterparts (p62^+/+^) providing further evidence that p62 expression depletes nuclear TDP-43 by shifting TDP-43 into the cytoplasm. It is not clear exactly how this occurs however, p62 may interact with TDP-43 via its PB1 domain, and/or recognize ubiquitinated TDP-43 via its UBA domain, thereby inducing mislocalisation and aggregation.

It is unclear whether the ALS and FTLD pathology associated with TDP-43 is due to loss of nuclear function or gain of toxic cytoplasmic functions. We hypothesised that p62 overexpression, leading to TDP-43 nuclear depletion, may cause changes in TDP-43 mediated splicing events. We investigated the effects of we p62 overexpression in neuronal cells on TDP-43-mediated RNA regulation. Loss of functional TDP-43 within the nucleus has previously been reported to induce the retention of exon 30b within the *STAG2* mature RNA^35^, exon 3 skipping from *POLDIP3*^36^ and regulation of *MADD*^35^ in human neuronal cells. We amplified *Stag2*, *Poldip3*, and *Madd* transcripts from RNA extracted from NSC-34 cells transfected with the p62 overexpression plasmid or an empty vector control, at 1, 3 or 5-days post transfection. Consistent with TDP-43 knockdown in previous reports, there was an increase in *Stag2* transcripts containing exon 30b in the p62 transfected cells after 3 and 5 days, compared with the empty vector transfected cells. Additionally, we observed a decrease in the full-length *Madd* transcript after 5 days. The moderate effect size that we observed may be due to low transfection efficiency in NSC-34 cells (~ 20–30%), such that only a subset of the total RNA extracted is from cells overexpressing p62. We found, in contrast to previous reports, a decrease in the number of transcripts skipping exon 3 (Δ3) in *Poldip3* following p62 overexpression compared with empty vector transfected cells at 5 days. Nonetheless, our findings are interesting as while changes in regulation of *Stag2* and *Madd* transcripts following p62 overexpression supports a loss of functional nuclear TDP-43, the splicing of *Poldip3* in mouse neurons was inconsistent with a previous report^36^. This may be due to differential splicing patterns and regulation between species. In support of this, TDP-43 regulation of *STMN2* cryptic splicing in ALS patient neurons was not observed in the mouse^37^.

In order to assess the effects of p62 overexpression on TDP-43 aggregation and possible toxic cytoplasmic gain of function we performed cell fractionation studies after co-expressing p62 (wild type or UBA deficient, ΔUBA) with TDP-43 and found that p62 wild type overexpression reduced the amount of soluble TDP-43 and increased the soluble:insoluble ratio. This suggests that expression of p62 may either induce TDP-43 degradation, thereby decreasing the soluble TDP-43 observed, or p62 may shift TDP-43 expression into insoluble protein aggregates. It appears to be the latter, as the total amount of wild type TDP-43 (combined soluble and insoluble, full-length and cleaved) was unchanged in cells overexpressing EGFP-p62 compared with cells expressing EGFP. Taken together, our experiments suggest that p62 is shifting TDP-43 localisation from a soluble nuclear fraction into an insoluble cytoplasmic fraction, rather than inducing degradation of TDP-43. To investigate this further we performed experiments to determine the effects of p62 overexpression on TDP-43 turnover. We treated cells with a proteasome inhibitor (MG132), serum starvation (to induce autophagy) or serum starvation with Bafilomycin treatment (to inhibit lysosomal degradation) followed by cell fractionation into soluble and insoluble fractions. In keeping with our previous observations, these studies did not reveal significant changes in the amount of total TDP-43 (soluble and insoluble combined) in cells over-expressing EGFP-p62 compared with EGFP expressing cells. However, a previous report has shown that p62 expression induced TDP-43 degradation^33^. It may be that the time frame of our experiments monitoring TDP-43 turnover was insufficient to observe the effects of p62 expression on TDP-43 degradation.

In addition to cytoplasmic mislocalisation and aggregation, TDP-43 cleavage is associated with ALS and FTLD pathology^38^. We observed that p62 overexpression induced TDP-43-tdTomato cleavage into a 90-kDa fragment. Given that the tdTomato tag is C-terminal and is ~ 55-kDa, this means the fragment corresponds to a C-terminal TDP-43 protein of ~ 35-kDa. Our TDP-43 turnover experiments revealed that this cleaved TDP-43 fragment was also produced in control EGFP-expressing cells, but only following treatment with a proteasome inhibitor, whereas this fragment was observed in lysates from cells expressing EGFP-p62 under all treatment conditions including in the lysates from untreated cells. Thus, proteasome inhibition and overexpression of EGFP-p62 lead to TDP-43 cleavage. Importantly a C-terminal 35-kDa fragment has been identified in patients and is associated with aggregation and loss of function^32,38^. Thus p62-induced cleavage of TDP-43 may be important in ALS and FTLD pathology. We excised the bands containing cleaved TDP-43 from our control, MG132 treated cells and the bands from untreated cells expressing p62 and subjected them to tryptic digestion and mass spectrometry. We determined that the fragment corresponds to amino acids 85–414 of TDP-43.

We performed a literature search of TDP-43 cleavage and found that both calpain and caspase-3 can lead to TDP-43 cleavage to a 35-kDa fragment^39,40^. The cleavage sites for calpain is in the C-terminus of TDP-43, and we determined by mass spectrometry that our cleavage site is in the N-terminus, thus it seemed unlikely that the TDP-35 fragment observed is mediated by calpain cleavage. TDP-43 contains a Caspase-3 dependent “DETD” recognition site, with cleavage occurring between amino acids 84–85^41^. Thus, we investigated whether p62-mediated cleavage of TDP-43 was dependent on calpain or caspases, using specific inhibitors. The amount of the cleavage product, TDP-35-tdTomato, in cells expressing EGFP-p62 was not significantly different in lysates from caspase inhibitor treated cells compared with untreated cells. However, in EGFP-p62 expressing cells treated with a calpain inhibitor the amount of TDP-35-tdTomato observed in the insoluble fraction was reduced compared to non-treated cells expressing EGFP-p62. This could suggest that the TDP-43 cleavage that is induced by p62 overexpression is partly dependent on calpains, but not caspases, however it is more likely a reflection of the reduced total TDP-43 that we observed in calpain-treated cells. It is possible that pre-treatment with the calpain inhibitor prior to transfections reduced the transfection efficiency thereby reducing the total TDP-43 and the TDP-35 fragment observed. Thus, we interpret our results to be consistent with the notion that p62-mediated TDP-43 cleavage occurs via another mechanism that is independent of both caspases and calpains.

Our results show that p62 overexpression induced signs of TDP-43 pathology and we confirmed an important role for p62 in TDP-43 localisation using p62^−/−^ MEFs, which showed significantly increased nuclear TDP-43 when compared with p62^+/+^ MEFs. As p62 is a stress-induced gene we hypothesise that stressors associated ALS, such as cigarette smoking or exposure to pesticides^42^, or proteasomal or oxidative stress^43,44^ may induce p62 expression and thereby lead to TDP-43 pathology. Here, we show that proteasome inhibition and exposure to the heavy metal arsenite induced p62 expression and this was associated with increased levels of cytoplasmic TDP-43. We also show that p62 overexpression induced neuronal death. However, we found that this cell death was independent of TDP-43 expression as knock-down of TDP-43 did not significantly reduce the cell death observed. Thus, while increased levels of p62 leads to TDP-43 mislocalisation and aggregation and misregulation of RNA transcripts in neurons, p62 appears to induce cell death via additional TDP-43 independent pathways.

### Concluding remarks

Our results show that p62-induced cleavage of TDP-43 requires the PB1 domain, the UBA domain and the nuclear export sequence, whereas mislocalisation required the PB1 and UBA domains only. Thus, cytoplasmic mislocalisation was not necessarily required for TDP-43 cleavage to occur. Importantly, our results show that overexpression of p62 that is lacking either the PB1 or the UBA domains does not induce TDP-43 cleavage or mislocalisation. Of note, p62 overexpression exacerbates disease in a SOD1 mouse model of ALS and here we have shown that p62 overexpression leads to altered TDP-43-mediated RNA regulation consistent with reduced TDP-43 nuclear function. Thus, modulating p62 expression may present a potential therapeutic target for ALS and FTD-TDP. We are currently investigating the use of phosphorodiamidate morpholino oligomers to regulate the expression of p62. Determination of the effects of p62 modulation on TDP-43 pathology in ALS patient-derived neuronal cells is the subject of our future research.

## Methods

### Cell lines and treatments

All experiments described were carried out in NSC-34 motor neuron-like cells or mouse embryonic fibroblasts (MEFs). Wild type (p62^+/+^) and p62 knockout (p62^−/−^) MEFs were a generous gift from Professors Yu-shin Sou and Masaaki Komatsu, described previously^45^. NSC-34 cells were maintained in Dulbecco’s modified essential medium (DMEM)/F12-Ham supplemented with 10% foetal calf serum in a 37 °C incubator with 5% CO_2_. MEF cells were maintained in DMEM with 10% foetal calf serum. Chemical treatments were purchased from Sigma-Aldrich. Cells were incubated overnight with 2 μM MG132, 10 μM sodium arsenite or were heat shocked at 42 °C for 1 h, followed by 2 h recovery at 37 °C. For inhibitor assays, cells were pre-treated for 1 h with either 20 μM caspase inhibitor (Promega) or 5 calpain inhibitor (Sigma-Aldrich) or left untreated prior to transfection. Cells were maintained in media containing the inhibitors for 48 h post-transfection. Cells were then fractionated into soluble and insoluble fractions and subjected to SDS polyacrylamide electrophoresis and western blotting.

### Plasmids and transfections

Cells were transfected with constructs as indicated for 6–24 h. The pcDNA3.1, pcDNA3.1/EGFP, pcDNA3.1/EGFP-p62 (wild type and UBA deficient) were described previously^46^. p62 deletion constructs were a kind gift from Professor Terje Johansen^47^. pcDNA3.1/TDP-43-tdTomato constructs were supplied by Professor Justin Yerbury. For soluble/insoluble fractionation experiments cells were grown in 6-well plates and transfected with 1.25 μg of pcDNA3.1/EGFP or pcDNA3.1/EGFP-p62 and 1.25 μg pcDNA3.1/TDP-43-tdTomato unless otherwise indicated. For experiments investigating the effects of increasing p62 on TDP-43 mislocalisation and cleavage, cells were grown in 6-well plates and transfected with 0.25, 0.5, 1, 1.5 or 2 μg of pcDNA3.1/EGFP-p62 and adjusted with 1.75, 1.5, 1, 0.5 or 0 μg of pcDNA3.1/EGFP respectively. All wells were also transfected with 1 μg pcDNA3.1/TDP-43-tdTomato. For transfection of cells with PMOs routinely used for knock-down of TDP-43 in our laboratory or a Genetools standard control (GTC), 1 × 10^6^ cells were resuspended in Buffer R. PMO was added at 100 μM. Cells were electroporated using the neon transfection tips as per manufacturer’s instructions (Invitrogen). The following day cells were transfected with 2 μg expression plasmids for either EGFP or EGFP-p62 and 48 h later were assessed for cell viability with Sytox Red by flow cytometry.

### Cell fractionation (nuclear:cytoplasmic)

p62^+/+^ or p62^−/−^ MEFs were fractionated following cell treatments. Briefly, cells were lysed with 500 µL fractionation buffer (10 mM HEPES (4-2-hydroxyethyl-1-piperazineethanesulfonic acid), 1.5 mM MgCl_2_, 10 mM KCl, 0.5 μM dithiothreitol, 0.05% Tergitol, pH 7.9) on ice for 10 min. Lysates were clarified by centrifugation at 3000 rpm at 4 °C for 10 min. Cytosolic supernatants were collected and frozen. The pellet was resuspended in 374 μL Buffer B (5 mM HEPES, 1.5 mM MgCl_2_, 0.2 mM Ethylenediaminetetraacetic acid, 0.5 mM dithiothreitol, 26% Glycerol (v/v), pH 7.9) and 26 μL 4.6 M NaCl then passed through a 25-guage needle 10 times and then incubated on ice for 30 min. The lysates were centrifuged at 15,000 rpm at 4℃ for 30 min. Nuclear supernatants were collected.

### Soluble and insoluble protein fractionation

NSC-34 cells were seeded into T25 cm^2^ flasks. The following day cells were transfected with an expression plasmid for TDP-43-tdTomato and either pcDNA3.1/EGFP, pcDNA3.1/EGFP-p62 wild type [WT] or deletion constructs as indicated. Forty-eight hours post-tranfection cells were lysed on ice with 1% Triton X-100 in phosphate buffered saline (PBS) supplemented with complete EDTA-free protease and phosphatase inhibitor cocktails. Lysates were freeze/thawed and then clarified by centrifugation at 15,000*g* for 30 min at 4 °C and the 1% Triton X-100 soluble fractions were collected. The pellets were subsequently washed four times with 1% Triton X-100 in PBS and Triton-X insoluble proteins were solubilized with the addition of 1% SDS and incubated for 1 h at 60 °C. Triton X-100-insoluble fractions were collected following centrifugation at 15,000*g* for 30 min at 4 °C.

### Western blot analyses

Protein samples were electrophoresed through SDS polyacrylamide gels and transferred to nitrocellulose membranes. Western blot analyses were performed as follows: membranes were blocked with 5% skim milk powder (SMP) in Tris-Buffered Saline (TBS) for 1 h at room temperature, membranes were then incubated at room temperature with primary antibody as follows: anti-RFP (ThermoFisher) at a dilution of 1:1000 in 5% BSA in TBS-T for 2 h, anti-GFP (ThermoFisher) at a dilution of 1:5000 in 5% SMP in TBS with 0.05% Tween-20 (TBS-T) for 1 h, or anti-α-tubulin (Sigma-Aldrich) at a dilution of 1:10,000 in 5% SMP in TBS-T for 1 h. Membranes were washed 3× with TBS-T and then incubated with a species appropriate horseradish peroxidase conjugated secondary antibody for 1 h at room temperature in 3% SMP in TBS-T. Membranes were washed 3× with TBS-T and protein bands visualised with enhanced Chemi-luminescence Plus reagent (Perkin Elmer). Membranes were stripped between western blots using mild stripping buffer (200 mM glycine, 3.5 mM sodium dodecyl sulphate, 0.2% Tween-20) and checked for residual signal with Chemiluminescence reagent (Perkin Elmer).

### Densitometry

Densitometry of bands from Western blot analyses was performed using the in-built analysis function in the BioRad ImageLab software. Data presented has been corrected to α-tubulin expression within samples and depicts the mean of ≥ 3 independent experiments +/− SEM.

### Cell viability

For assessment of cell viability, NSC-34 cells were seeded into T25 cm^2^ flasks. Cells were transfected at various time points to allow for simultaneous assessment of viability at the following times post-transfection; 96, 72, and 48 h. Cells were transfected with pcDNA3.1/EGFP-p62 (wild-type) and TDP-43-tdTomato or suitable tagged-empty vector control. Cells were washed twice with 1 mL ice-cold PBS and transferred to 1.5 mL microfuge tubes and centrifuged at 1000 rpm for 2 min. Cells were resuspended in 200 µL ice-cold PBS + 2% FBS. A positive control sample was prepared by taking half of an untransfected sample and heating it at 65 °C for 1 min, followed by a 1 min incubation on ice. Half of the “killed” sample was resuspended in an equivalent volume of the remaining untransfected sample.

Samples were transferred to tubes and 0.2 µL SYTOX Red stain added to each sample that were then gently mixed and incubated at room temperature, shielded from light, for a minimum of 15 min. Samples were then analysed on a Fortessa flow cytometer, with 50,000 events collected per sample. A population of cells co-expressing EGFP-p62 and TDP-43-tdTomato was created, and viability was assessed by measuring the amount of fluorescence in the SYTOX Red channel. SYTOX Red is an exclusion dye and only permeates the membrane of dead cells, therefore populations with higher fluorescence were considered non-viable. The kill control sample was used to define the gating parameters for the viable vs dead/non-viable cell population. For assessment of the effect of TDP-43 knockdown on p62 overexpression induced cytotoxicity, cells were electroporated with PMOs to knockdown TDP-43 expression or a PMO control and plated in a 6-well plate. The following day cells were transfected with 2 μg EGFP or EGFP-p62. Forty-eight hours later cells were collected and stained with Sytox Red then assessed by flow cytometry as described.

### In-gel trypsin digestion and liquid chromatography mass spectrometry (LC–MS)

Triton-X insoluble fractions from cells transfected with TDP-43-tdTomato and either (i) pcDNA3.1 empty vector (cells were treated overnight with 10 μM MG132) or (ii) EGFP-p62^WT^transfected (cells were left untreated) cells were separated in duplicate through 10% SDS polyacrylamide gels. One gel was Coomassie stained and destained in 7% acetic acid, 10% methanol while the second gel was transferred to nitrocellulose membrane and western blot analysis for TDP-43-tdTomato (anti-red fluorescence protein, RFP) was performed. Bands of ~ 90 kDa in the Coomassie stained gel were identified by comparison with the molecular weight of the TDP-43-tdTomato from the western blot results. The bands in the gel were excised and cut into 1–2 mm pieces and destained in 50 mM ammonium bicarbonate pH 8, followed by 50 mM ammonium bicarbonate/50% acetonitrile pH 8. The destained gel pieces were dehydrated in 100% acetonitrile and the solution was removed to allow gel pieces to air dry. The proteins were reduced and alkylated with 10 mM DTT and 20 mM iodoacetamide (IAA) respectively and digested with trypsin (12.5 ng/µL) overnight at 37 °C as described^48^. Following overnight digestion, the supernatant was transferred to a fresh tube and the tryptic peptides from the gel pieces were extracted twice with 50% acetonitrile/2% formic acid and combined with the supernatants. The tryptic peptides were vacuum centrifuged to remove acetonitrile and desalted on a pre-equilibrated C_18_ Omix tip. The eluted peptides were further dried under vacuum centrifugation until ~ 10 µL remained for LC–MS/MS analysis.

Tryptic peptides were separated on a Ultimate 3000 (ThermoFisher) equipped with a Thermo Acclaim™ PepMap™ 100 C_18_ column (75 µm diameter, 3 µm particle size, 150 mm length) employing a 60 min gradient (2–26% v/v acetonitrile, 0.1% v/v formic acid for 40 min followed by 50% v/v acetonitrile, 0.1% v/v formic acid for 10 min and 80% v/v acetonitrile, 0.1% v/v formic acid for 8 min) with a flow rate of 300 nL/min. The peptides were eluted and ionized into Q-Exactive Plus mass spectrometer (ThermoFisher). The electrospray source was fitted with an emitter tip 10 μm (ThermoFisher) and maintained at 1.6 kV electrospray voltage. FTMS analysis was carried out with a 70,000 resolution and an AGC target of 1 × 10^6^ ions in full MS (m/z range 400–2000); and MS/MS scans were carried out at 17,500 resolution with an AGC target of 2 × 10^4^ ions. Maximum injection times were set to 30 and 50 ms respectively. A top-10 method was employed for MS/MS selection, ion selection threshold for triggering MS/MS fragmentation was set to 1 × 10^4^ counts, isolation width of 2.0 Da, and dynamic exclusion for 20 s was used to perform HCD fragmentation with normalised collision energy of 27.

Raw spectra files were processed using the Proteome Discoverer 2.2 software (ThermoFisher) incorporating the Sequest search algorithm. Peptide identifications were determined using a 20-ppm precursor ion tolerance and a 0.1 Da MS/MS fragment ion tolerance for FTMS and HCD fragmentation. Carbamidomethylation modification of cysteines was considered a static modification while oxidation of methionine, and acetyl modification on N-terminal residues were set as variable modifications allowing for maximum two missed cleavages. The data was processed through Percolator for estimation of false discovery rates. Protein identifications were validated employing a q-value of 0.01.

### Confocal microscopy

To enable separation of single cells for better imaging, cells were transfected and then reseeded onto coverslips the following day. Briefly, NSC-34 cells were transfected in 6-well plates with expression vectors for TDP-43-tdTomato (wild type [WT] or mutant [p.M337V] and either pcDNA3.1-EGFP (empty vector [EV]), pcDNA3.1-EGFP p62 (WT or deletion constructs as indicated). The following day cells were trypsinised and reseeded onto Poly-L-Lysine coated coverslips in 6-well plates. The following day 48 h post-transfection cells were washed 2 times with PBS and then fixed with 4% paraformaldehyde in PBS for 20 min, followed by washing 3 times with PBS. Coverslips were incubated with Hoechst 33342 (ThermoFisher) dye diluted 1:10,000 in PBS for 10 min at room temperature. Cells were washed 3 times with PBS and the coverslips mounted onto slides using Prolong Gold antifade mounting media (ThermoFisher). Images were taken using a Nikon A1 confocal microscope using a 40× oil objective. Pearson’s co-efficient to determine the co-localisation of TDP-43-tdTomato with Hoechst nuclear stain was performed using the co-loc 2 plug in for ImageJ on cell images taken from 3 independent replicate experiments (minimum 32 cells per transfection). A Pearson’s co-efficient of > 0.6 indicates co-localisation.

### RNA extraction and RT-PRC

NSC-34 cells were transfected with p62 overexpression plasmids or an empty vector, as above, and incubated for 1, 3 or 5 days prior to harvesting for transcript analysis. RNA was isolated from cells using a MagMAX total RNA isolation kit, including a DNase treatment (Life Technologies), performed using a Kingfisher RNA extractor, according to the manufacturer’s protocol. RT-PCRs were performed using a One-step SuperScript III RT-PCR kit with platinum Taq polymerase (Life Technologies), according the manufacturer’s instructions. Each reaction contained 50 ng of RNA and 25 ng of each forward and reverse primer, with the primer sequences and PCR cycling conditions listed in Table 2. PCR products were separated on a 2% agarose gel, stained using RedSafe (Scientifix) and visualised on a Vilber Lourmat Fusion FX system. Densitometry was performed using Bio1D software.

**Table 2:** Primer sequences and RT-PCR conditions for *Stag2*, *Poldip3*, *Madd* and *Smn* transcript amplification.

Transcript and size	Primer	Primer sequence	Temperature profile	
*Stag2*	Exon 29 Fwd	GGCATGCAACTAGCACTCC	55 °C 30 min	
+ 30b 346 bp	Exon 32 Rev	CTTCCATTAGGCTTGTACCACG	94 °C 2 min	
Δ30b 235 bp			94 °C 40 s	
			55 °C 30 s	28 cycles
			68 °C 1 min	
*Poldip3*	Exon 2 Fwd	CCGGTTTCGGATCAAAGGGA	55 °C 30 min	
FL 349 bp	Exon 4 Rev	CATCTGCTTGGGAGGGACAG	94 °C 2 min	
Δ3 260 bp			94 °C 40 s	
			55 °C 30 s	30 cycles
			68 °C 1 min	
*Madd*	Exon 27 Fwd	CTGCGTGGTGTTGCGTAGTA	55 °C 30 min	
FL 349 bp	Exon 30/31	GAAAACCTTCCGCTCCTGGTTG	94 °C 2 min	
	Rev		94 °C 40 s	
			55 °C 30 s	32 cycles
			68 °C 1 min	
*Smn*	Exon 4 Fwd	GAAAGTCAAGTTTCCACAGACG	55 °C 30 min	
FL 430 bp	Exon 8 Rev	CACCCCATCTCCTGAGACAGAGC	94 °C 2 min	
			94 °C 40 s	
			60 °C 30 s	28 cycles
			68 °C 1 min	

### Statistical analyses

All statistical analyses were performed in SPSS Version 24.0.0.0. The significance of differences between means was determined using the One-way analysis of variance with post-hoc Bonferroni test with significance set at *p* ≤ 0.05.

## Supplementary Information

Supplementary Figures.

## Abbreviations

ALS: Amyotrophic lateral sclerosis
FTLD: Frontotemporal lobar degeneration
PB1: Phox and bemp1
UBA: Ubiquitin-associated
NF-κB: Nuclear factor kappa b
SOD1: Superoxide dismutase 1
MEFs: Mouse embryonic fibroblasts
DMEM: Dulbecco’s modified essential medium
EGFP: Enhanced Green Fluorescent Protein
PBS: Phosphate buffered saline
LC–MS: Liquid Chromatography Mass spectrometry
NaAsO_2_: Sodium arsenite
