# Supplementary material for: p62 overexpression induces TDP-43 cytoplasmic mislocalisation, aggregation and cleavage and neuronal death
Source: Sci Rep. 2021 Jun 1;11:11474. doi: 10.1038/s41598-021-90822-2 (PMC8169680; doi:10.1038/s41598-021-90822-2)
Supplement: Supplementary file 1 — Supplementary Figures. [file 41598_2021_90822_MOESM1_ESM.pdf]

p62 overexpression induces TDP-43 cytoplasmic mislocalisation, aggregation and cleavage and causes neuronal death

Supplementary Figures

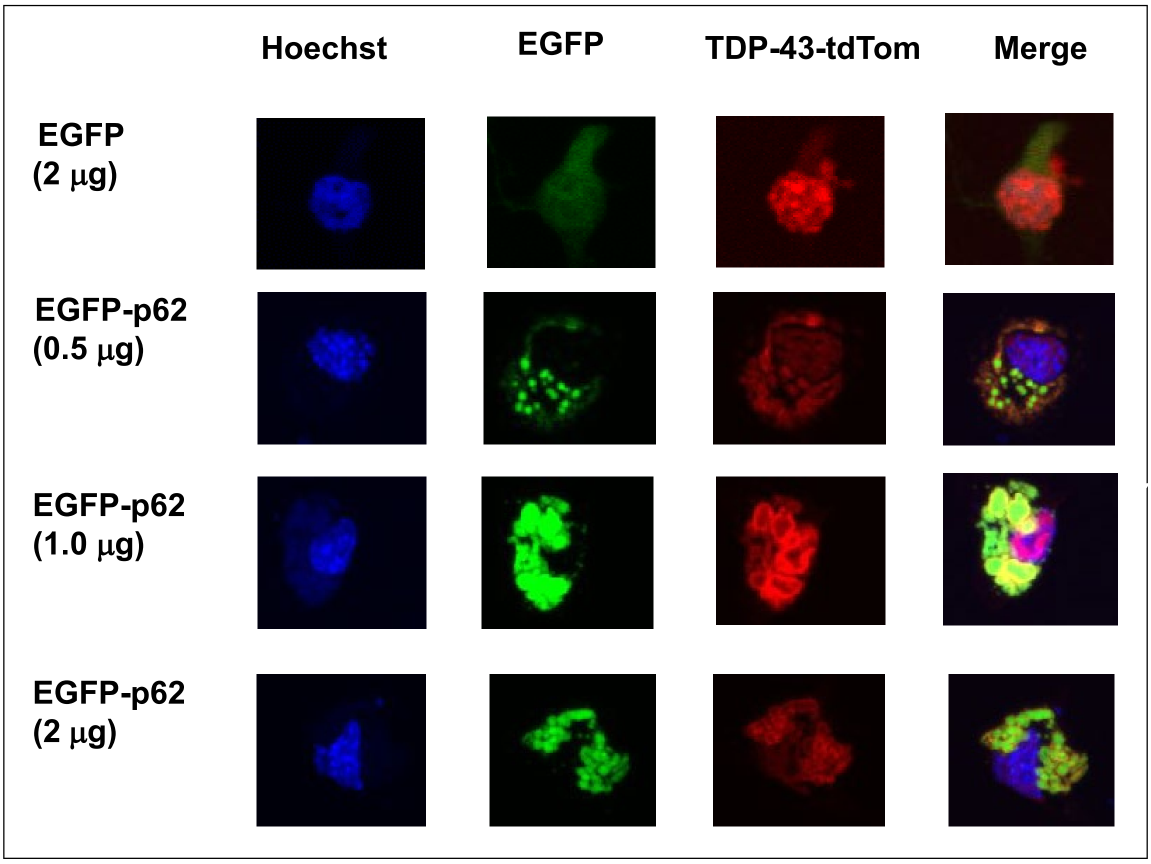

Supplementary Figure 1. Increasing p62 leads to increased nuclear depletion of TDP-43. NSC-34 cells were transfected with EGFP or increasing amounts (0.5, 1 or 2 µg) of EGFP-p62 and TDP-43-tdTomato. Image is representative of 3 independent experiments.

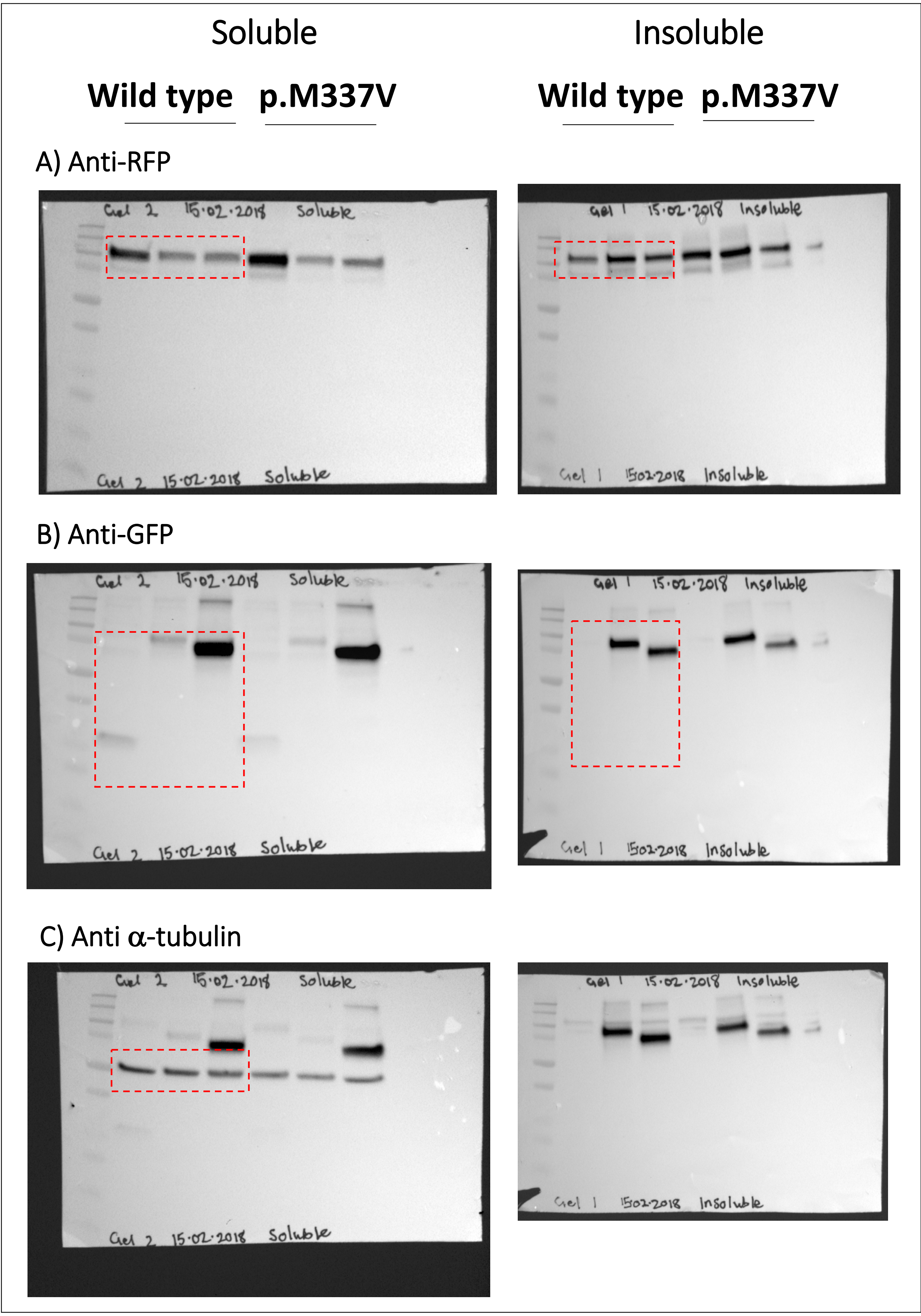

Supplementary Figure 2. p62 expression alters TDP-43 solubility - blot images. Lane order: Molecular weight marker, EV, EGFP-p62 wild type, EGFP-p62 dUBA – all with co-expressed TDP-43-tdTomato wild type, , EV, EGFP-p62 wild type, EGFP-p62 dUBA – all with co-expressed TDP-43-tdTomato p.M337V,. Please note Right hand side of blots depicting bands observed in TDP-43-tdTomato p.M337V were cropped out of final figures.

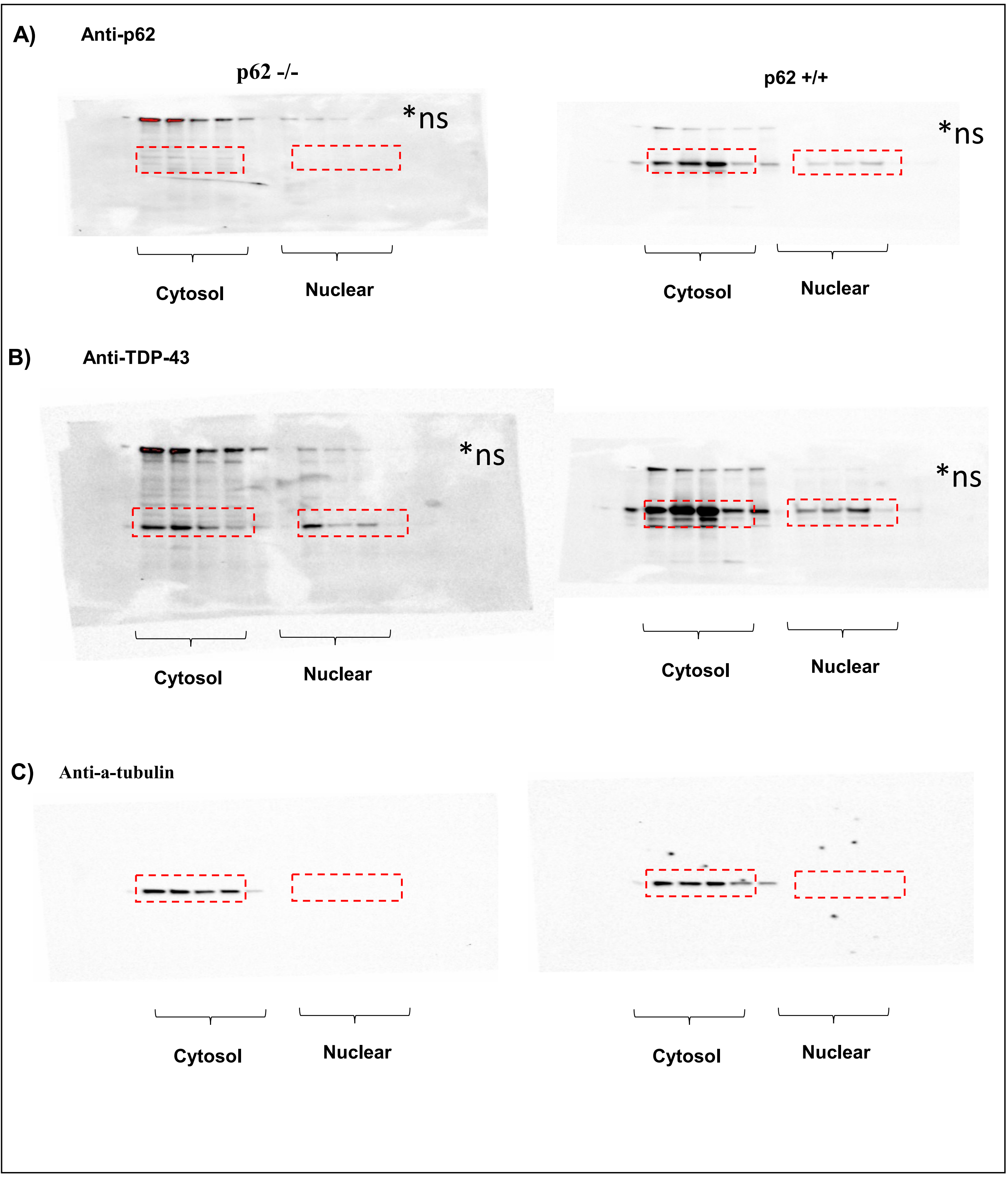

Supplementary Figure 3. p62 alters the nuclear:cytoplasmic TDP-43 ratio – blot images. Lane order Cytoplasmic: non-treated, MG132, AsNaO2, heat shock, Luperox (cropped out of final figure 3), nuclear fraction: non-treated, MG132, AsNaO2, heat shock, Luperox (cropped out of final figure 3). A) anti-p62, B) anti-TDP-43, C) anti-α-tubulin.

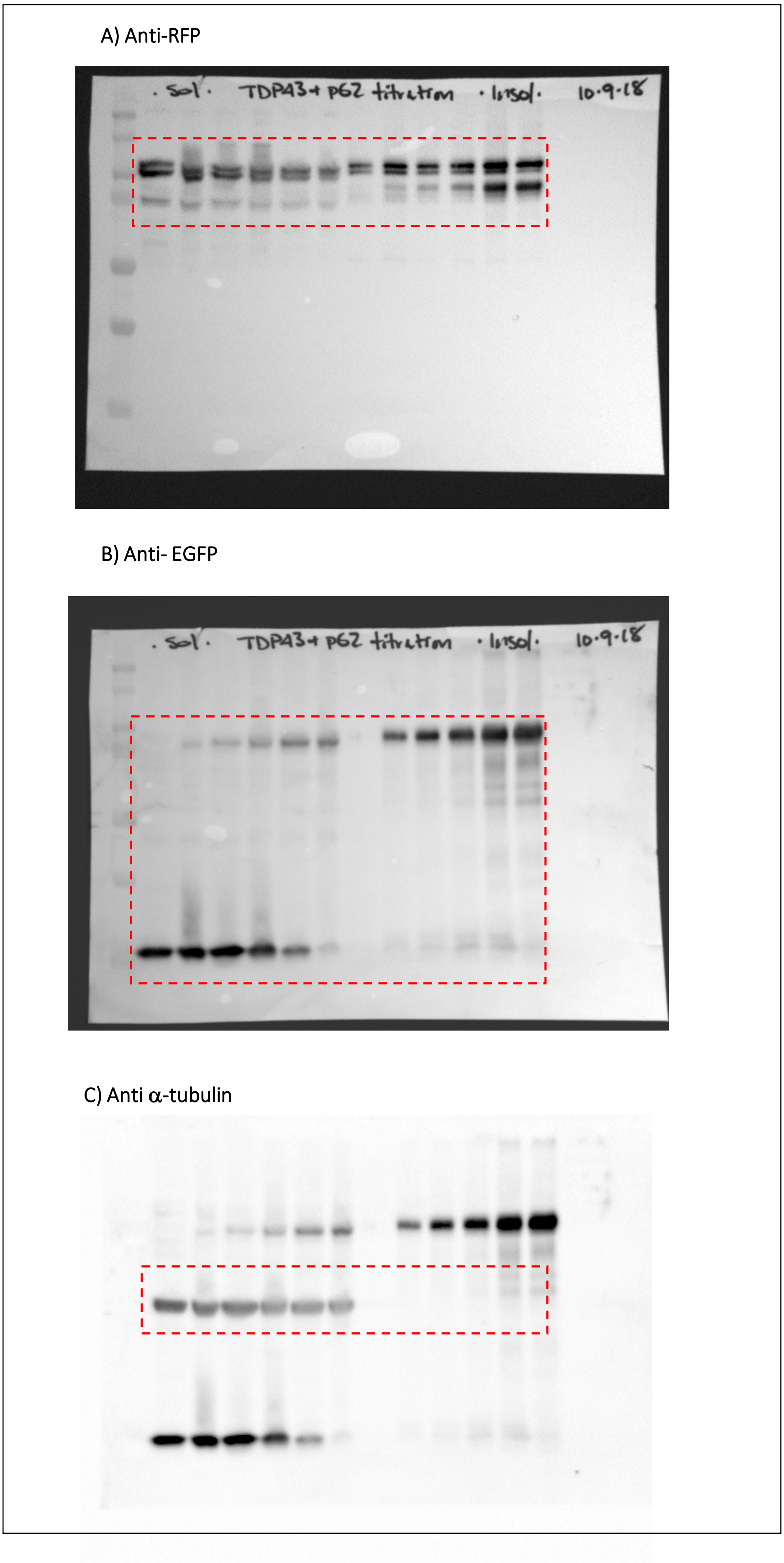

Supplementary Figure 4. p62 expression induces TDP-43 cleavage to a 35-kDa fragment - blot images. Lane order: soluble lysates from cells transfected with increasing amounts of EGFP-p62 (0, 0.25, 0.5, 1, 1.5 or 2 μg) and a corresponding decreasing amounts of EGFP (2, 1.75, 1.5, 1, 0.5 or 0 μg) along with a consistent amount of TDP-43-tdTomato, insoluble lysates in the same order. A) anti-RFP, B) anti-EGFP, C) anti-α-tubulin (not stripped after anti-EGFP).

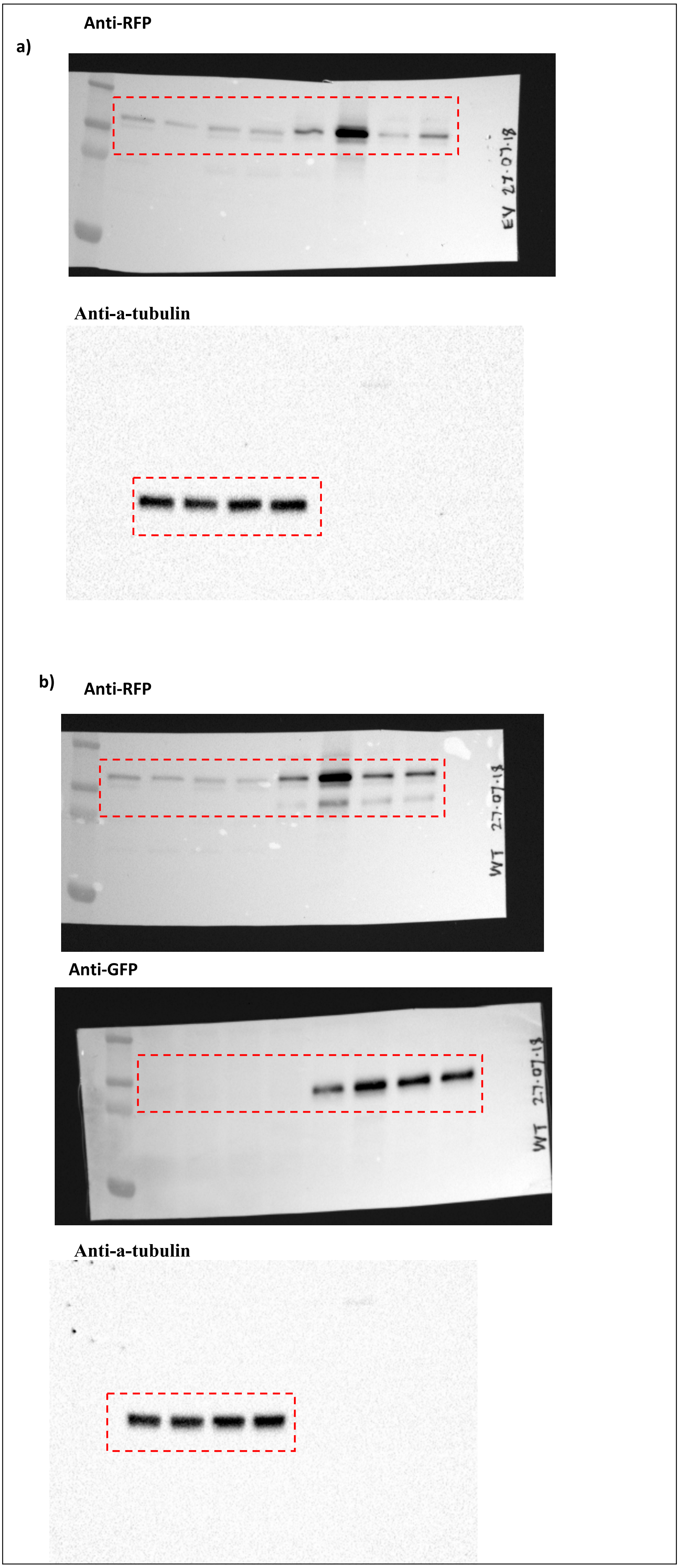

Supplementary Figure 5. p62 creates a cleaved TDP-43 species that is also induced by proteasomal inhibition. - blot images. A) Lane order: soluble fractions from cells transfected with pcDNA3.1 and TDP-43-tdTomato, non-treated, MG132, serum starved or serum starved and bafilomycin treated. Insoluble fractions from the same cells. B) Lane order: soluble fractions from cells transfected with EGFP-p62 and TDP-43-tdTomato, non-treated, MG132, serum starved or serum starved and bafilomycin treated. Insoluble fractions from the same cells. Anti-RFP (top panel), anti-a-tubulin (2nd panel), anti-RFP (3rd panel), anti-GFP (4th panel), anti-α-tubulin (bottom panel).

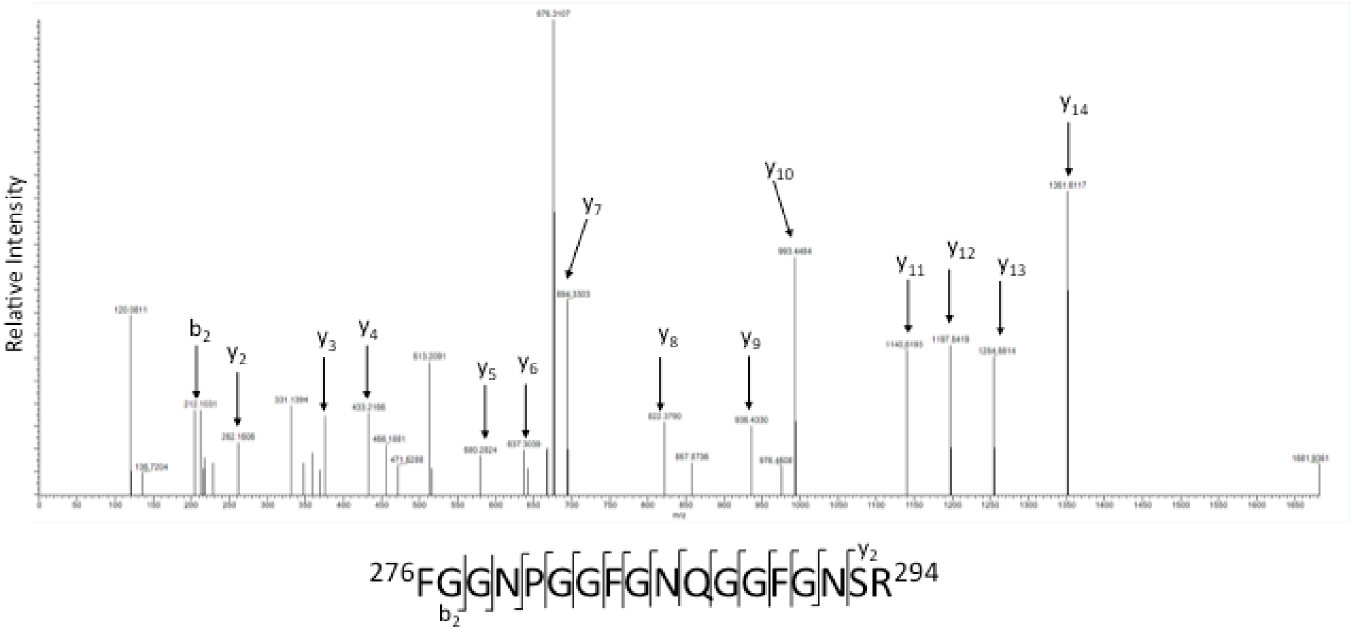

Supplementary Figure 6. MS/MS fragmentation for tryptic peptide ^{276}FGGNPGGFGNQGGFGNSR^{294} from 90 kDa tdTomato-TDP-43.

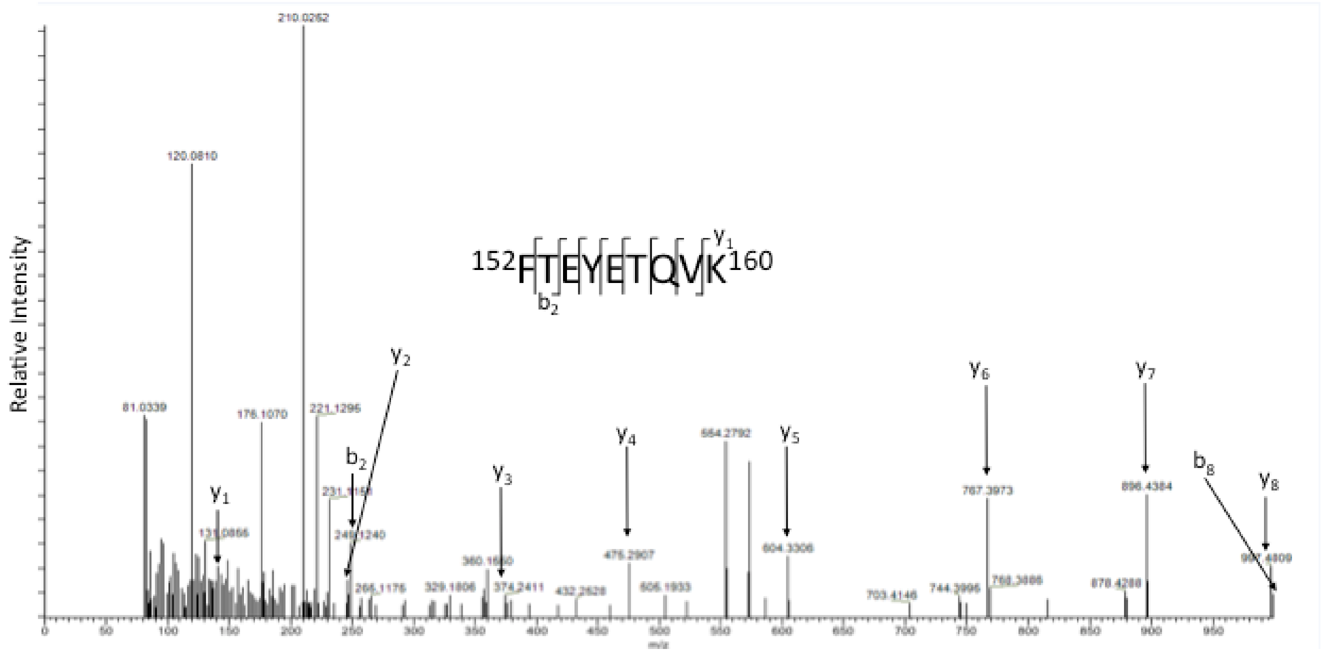

Supplementary Figure 7 . MS/MS fragmentation of tryptic peptide ^{152}FTEYETQVK^{160} from 90 kDa tdTomato-TDP-43.

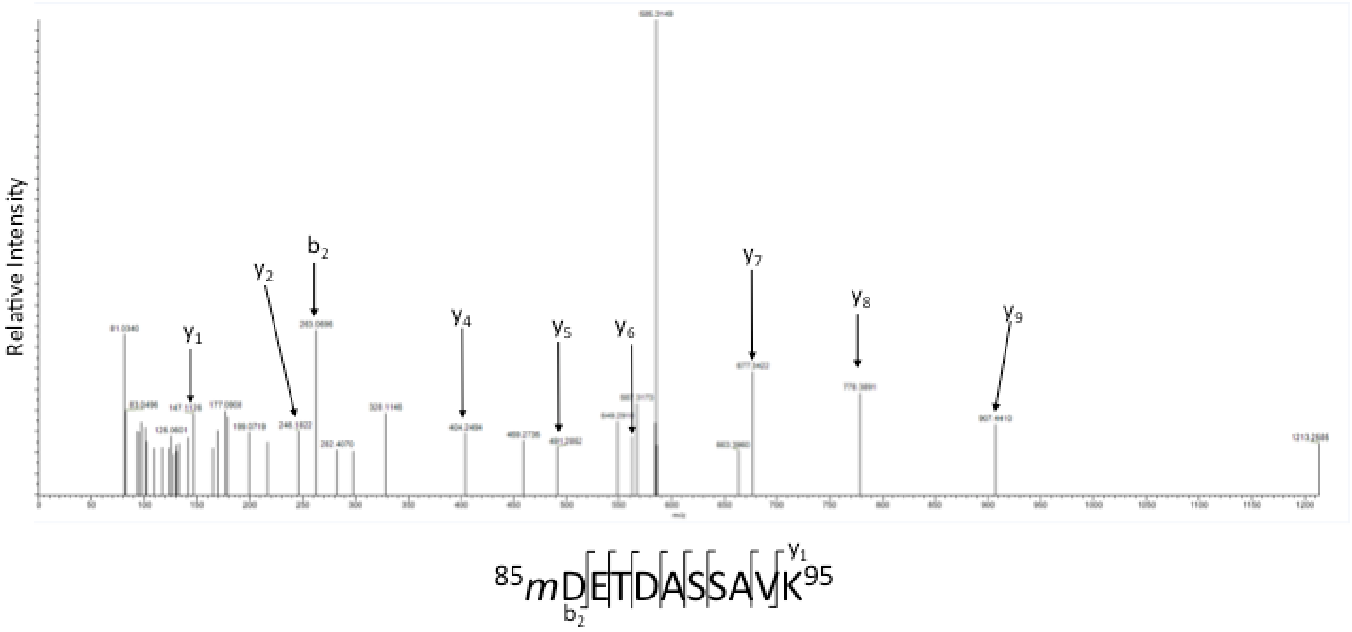

Supplementary Figure 8. MS/MS fragmentation of tryptic peptide
^{85}mDETDASSAVK^{95} from 90 kDa tdTomato-TDP-43.

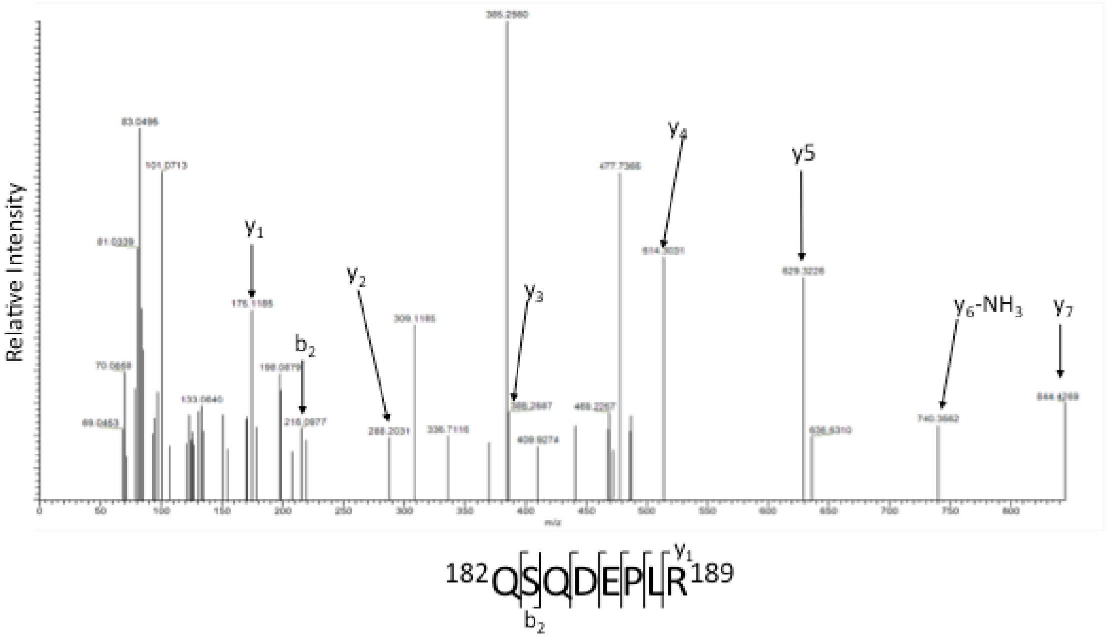

Supplementary Figure 9. MS/MS fragmentation of tryptic peptide [182]QSQDEPLR[189] from 90 kDa tdTomato-TDP-43.

Q13148 – TDP-43

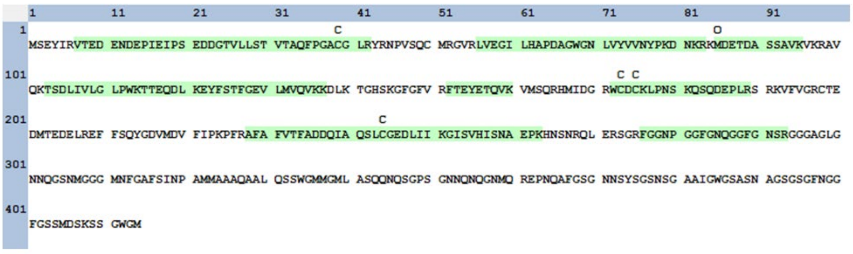

Supplementary Figure 10. LC-MS/MS analyses of a control HEK293 lysate to identify typical tryptic peptides from endogenous TDP-43 excised from a region on a protein gel ~37 kDa – 50 kDa, which contained coverage at the N-terminus of TDP-43.

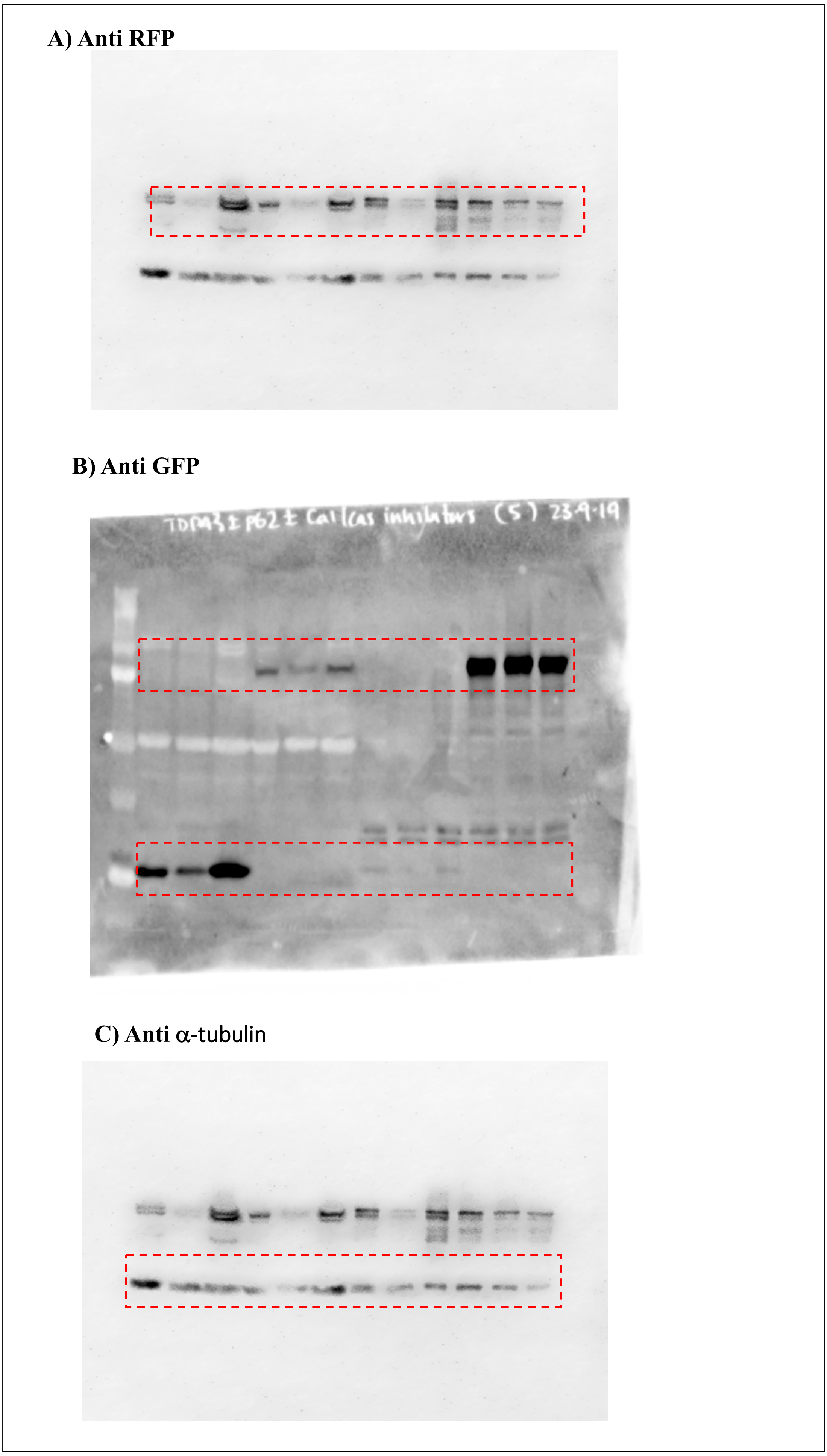

Supplementary Figure 11. p62-mediated cleavage of TDP-43 is independent of caspases – blot images. Lane order: soluble fractions from non-treated, caspase treated or calpain treated empty-vector transfected cells, soluble fractions from non-treated, caspase treated or calpain treated EGFP-p62 transfected cells, insoluble fractions in the same lane order. A) anti-RFP, B) anti-GFP, and C) anti-α-tubulin.

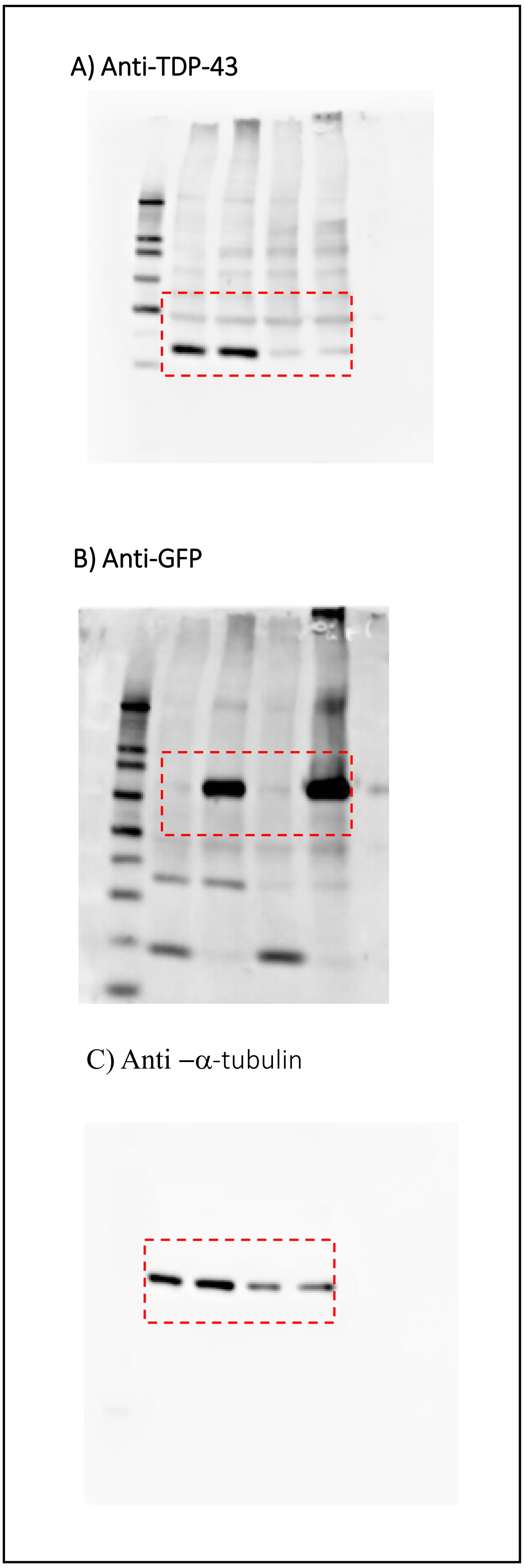

Supplementary Figure 12. p62-mediated neuronal death is independent of TDP-43 expression – blot images. Lane order: Lysates from cells transfected with pcDNA3.1 or EGFP-p62 and a genetools control, lysates from cells transfected with pcDNA3.1 or EGFP-p62 and a PMO to knockdown TDP-43 expression. A) anti-TDP-43, B) anti-GFP and C) anti-α-tubulin.

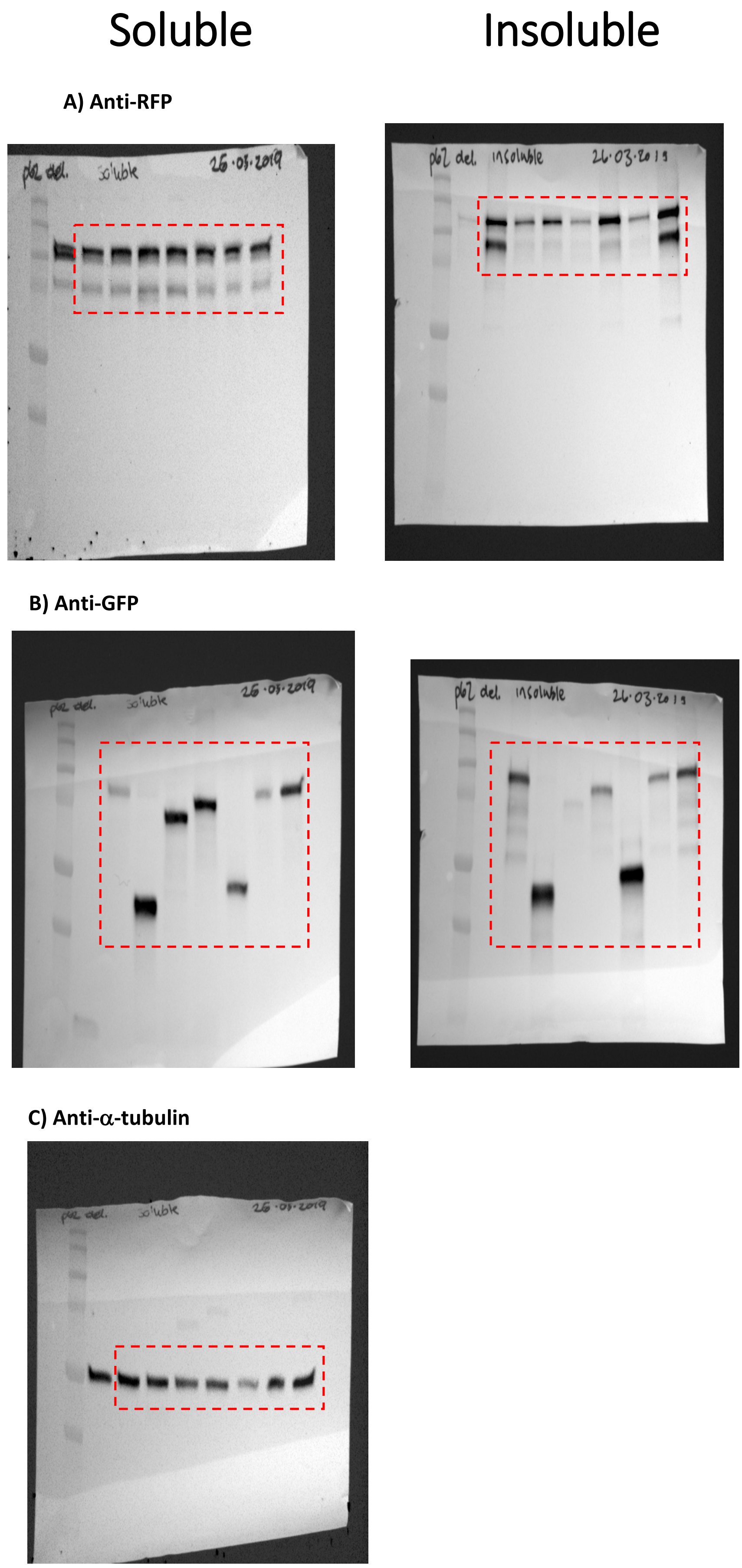

Supplementary Figure 13. TDP-43 aggregation and cleavage require the PB1 and UBA domains, and the nuclear export signal of p62 – blot images. Lane order: molecular weight marker, EGFP, EGFP-p62 wild type, EGFP-p62 PB1 only , EGFP-p62 ΔPB1, EGFP-p62 ΔUBA, EGFP-p62 PB1 and UBA only, EGFP-p62 ΔNES and EGFP-p62 K7A/D69A. A) anti-RFP (TDP-43), B) anti-GFP (p62) and C) anti-α-tubulin.

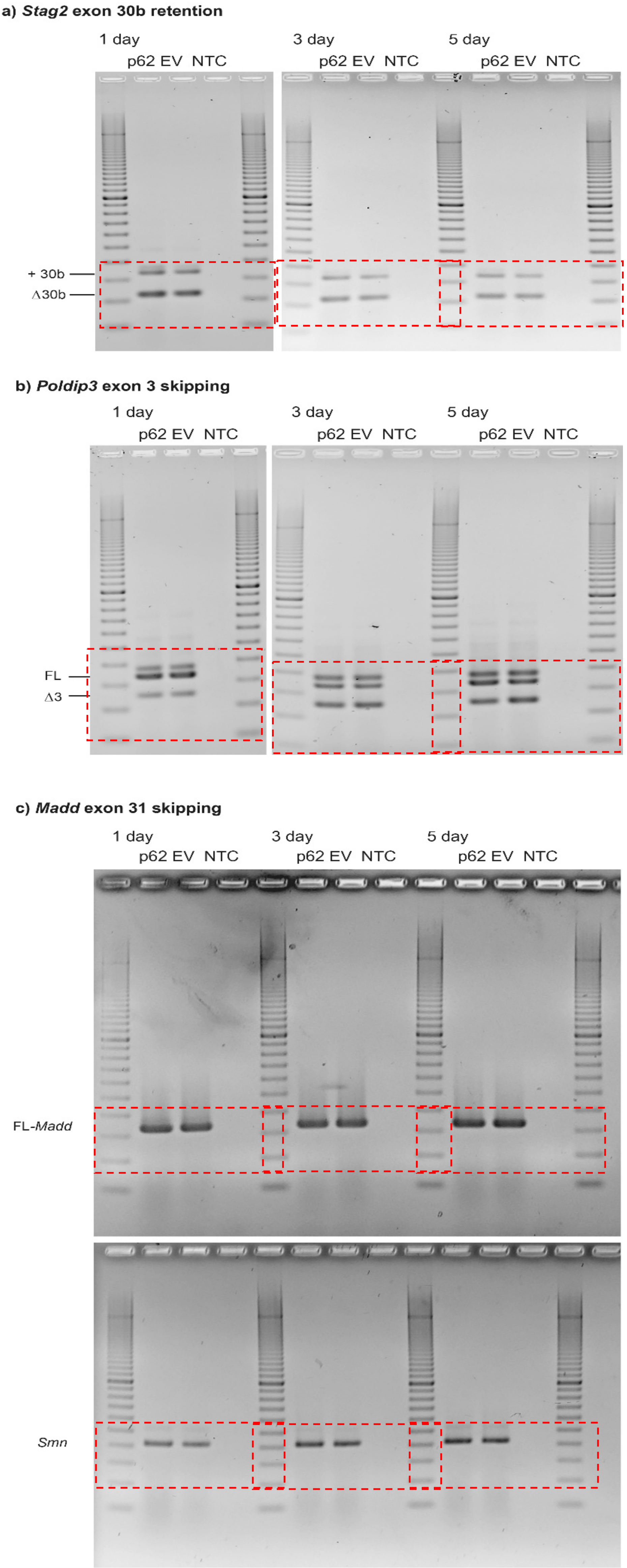

Supplementary Figure 14. p62 overexpression leads to loss of TDP-43-mediated RNA regulation– gel images. Lanes order as labelled. Please note that Day 1 PCRs for Stag2 and Poldip3 were run separately to Days 3 and 5. Images were subsequently cropped as shown for consistency within the final figure.
